# Uremic Toxin-Driven Vascular Calcification in Chronic Kidney Disease: Molecular Pathways and Integrated Phenotypes

**DOI:** 10.3390/toxins18020112

**Published:** 2026-02-21

**Authors:** Rodolfo Fernando Rivera, Maria Teresa Sciarrone Alibrandi, Nadia Edvige Foligno, Lorenza Magagnoli, Paola Ciceri, Mario Cozzolino

**Affiliations:** 1 Nephrology and Dialysis Unit, Pio XI Hospital ASST-Brianza, 20832 Desio, Italy; rodolfo.rivera@asst-brianza.it; 2 Nephrology and Dialysis Unit, IRCCS San Raffaele Scientific Institute, 20132 Milan, Italy; sciarronealibrandi.mariateresa@hsr.it (M.T.S.A.); foligno.nadia@hsr.it (N.E.F.); 3 Nephrology and Dialysis, Department of Health Sciences, University of Milan, 20142 Milan, Italy; lorenza.magagnoli@unimi.it (L.M.); paola.ciceri@unimi.it (P.C.)

**Keywords:** vascular calcification, chronic kidney disease, uremic toxins, phenotypic clusters, indoxyl sulfate, precision medicine, therapeutic targets

## Abstract

Background: Vascular calcification (VC) affects up to 90% of patients with end-stage renal disease and increases cardiovascular mortality 3- to 5-fold. Once considered passive mineral deposition, VC is now recognized as an active, toxin-driven process orchestrating vascular smooth muscle cell transdifferentiation, endothelial dysfunction, and matrix remodeling. However, current uremic toxin classifications remain biochemically oriented, providing limited clinical guidance for risk stratification and therapeutic selection. Methods: This comprehensive review reframes uremic toxin-driven VC through an integrated phenotypic lens, synthesizing molecular mechanisms, clinical biomarkers, and therapeutic targets into a unified translational framework. Results: We propose five mechanistic-clinical phenotypes representing distinct biological trajectories of vascular injury. These include (1) inflammatory-oxidative (dominated by indoxyl sulfate, p-cresyl sulfate, NLRP3 inflammasome activation), (2) mineral-metabolic (hyperphosphatemia, FGF23 excess, Klotho deficiency), (3) epigenetic-senescent (histone modifications, microRNA dysregulation, cellular senescence), (4) endocrine cross-talk (vitamin D, PTH, gut-derived metabolites), and (5) integrated toxic continuum (convergence of multiple pathways in advanced disease). A comprehensive biomarker panel spanning inflammatory markers, mineral metabolism parameters, epigenetic indicators, and endocrine-gut metabolites enables phenotypic stratification and therapeutic monitoring. Emerging therapies—including tissue-nonspecific alkaline phosphatase inhibition, ectonucleotide pyrophosphatase/phosphodiesterase 1 enzyme replacement, vitamin K_2_ activation, senolytic agents, and SNF472 crystal-growth blockade—are mapped to their optimal phenotypic contexts. Conclusions: This phenotype-oriented paradigm transforms VC from an inevitable complication into a targetable and potentially reversible manifestation of uremic toxicity, establishing a translational foundation for precision-based vascular medicine in chronic kidney disease. The framework enables biomarker-guided patient stratification, rational therapeutic selection, and phenotype-enriched clinical trial design.

## 1. Introduction

Vascular calcification (VC) represents one of the most characteristic and devastating manifestations of chronic kidney disease (CKD), affecting both large elastic arteries and smaller muscular vessels. Its prevalence increases steadily with declining renal function, reaching up to 90% in patients with end-stage renal disease (ESRD) [1]. Once regarded as a passive precipitation of calcium–phosphate (Ca-Pi) salts, VC is now recognized as an active, cell-mediated process governed by vascular smooth muscle cells (VSMCs), endothelial cells, inflammatory mediators, and profound disturbances in mineral metabolism [2,3].

Two main histopathological patterns are traditionally distinguished. Intimal calcification, associated with atherosclerotic lesions, develops within lipid-rich plaques characterized by macrophage infiltration and inflammatory instability [4,5]. In contrast, medial (Mönckeberg-type) calcification, which predominates in CKD, is largely non-atherosclerotic and reflects the phosphate-driven osteo-chondrogenic transdifferentiation of VSMCs, accompanied by extracellular matrix remodeling and elastin degradation [6,7].

Calcific degeneration may also extend to cardiac valves [8], particularly the aortic and mitral leaflets, where shared triggers such as hyperphosphatemia, oxidative stress, and Klotho deficiency lead to fibrotic thickening and progressive hemodynamic impairment [9].

Over the past decade, the understanding of VC has shifted from a static physicochemical model toward a multidimensional biological framework, in which phosphate toxicity, oxidative stress, and uremic toxin accumulation act as convergent drivers of vascular injury [10]. Phosphate—once viewed as a mere electrolyte—has emerged as a bona fide uremic toxin, capable of activating Runx2-dependent osteogenic programs and impairing endothelial nitric oxide (NO) signaling through oxidative stress [11]. Protein-bound solutes such as indoxyl sulfate (IS) and p-cresyl sulfate (PCS) further amplify phosphate-induced calcification via activation of the AhR–NF-κB and p38 MAPK pathways [12], impairing mitochondrial function and promoting apoptosis with the release of calcifying extracellular vesicles—recognized precursors of hydroxyapatite nucleation [2,3].

Additional tryptophan-derived metabolites, including kynurenine and indole-3-acetic acid, extend this toxic network by linking oxidative stress with endothelial-to-mesenchymal transition (EndMT) and vascular stiffness [13]. Collectively, these findings support the concept that VC is a toxic vascular phenotype—the morphological expression of systemic molecular stress, in which uremic solutes, oxidative cascades, and inflammatory circuits converge to remodel the vascular wall.

At the molecular level, uremic toxins disrupt redox homeostasis by activating NADPH oxidase and suppressing antioxidant enzymes such as superoxide dismutase (SOD) and catalase. They interfere with endothelial nitric oxide synthase (eNOS) activity, promote leukocyte adhesion, and accelerate endothelial senescence. In VSMCs, these compounds induce chromatin remodeling through histone acetylation and microRNA deregulation [14], thereby establishing a durable pro-calcific phenotype.

Importantly, the vascular response to uremic toxins is heterogeneous. Experimental and clinical observations indicate multiple toxin-driven phenotypes, reflecting differences in oxidative reactivity, hormonal cross-talk, and vascular resilience. Such diversity may explain why calcification progresses rapidly in some CKD patients but remains minimal in others with comparable biochemical parameters [15,16].

From a translational standpoint, VC in CKD has become a model of systemic uremic toxicity, extending its relevance beyond nephrology. The same toxins that drive vascular injury also contribute to neurological, immunologic, and metabolic dysfunction, reinforcing the view of uremia as a multisystem biological syndrome rather than a simple biochemical disorder. This recognition has prompted a shift from the physicochemical concept of “retained solutes” toward a phenotype-oriented paradigm, in which molecular complexity is interpreted through pathophysiologic and clinical manifestations.

Accordingly, this review provides an integrated perspective on VC in advanced CKD, merging toxicological, molecular, and therapeutic insights into a unified conceptual framework. We revisit the evolution of uremic toxin classification, tracing how conceptual refinements have led from chemical cataloguing to a multidimensional, clinically anchored model. Building upon this foundation, we propose a phenotypic interpretation of uremic toxin-driven vascular injury and outline the underlying promotive and inhibitory mechanisms that determine vascular resilience or vulnerability. Finally, we discuss current and emerging therapeutic approaches aimed at restoring vascular homeostasis and reducing the cardiovascular burden in CKD.

## 2. Methods

This comprehensive review synthesizes mechanistic and clinical evidence on uremic toxin-driven VC through narrative synthesis of the existing literature. We conducted a systematic literature search across PubMed, Scopus, and Web of Science, covering publications from 2010 to 2024. Search terms combined uremic toxins (“uremic toxins,” “indoxyl sulfate,” “p-cresyl sulfate,” “chronic kidney disease”), vascular calcification (“vascular calcification,” “arterial stiffness,” “medial calcification”), and mechanistic pathways.

We included peer-reviewed mechanistic studies, observational cohorts, and clinical trials examining uremic toxin-calcification relationships. Non-English publications and conference abstracts were excluded.

The proposed phenotypic classification emerged through iterative analysis of recurring patterns where specific toxin clusters consistently activated convergent pathological pathways across multiple independent studies. Each phenotype was characterized by: 1—biological plausibility from experimental models, 2—clinical observability through biomarker co-occurrence in published cohorts, and 3—therapeutic relevance with differential intervention responses.

This classification represents a hypothesis-generating conceptual framework requiring prospective validation, not an empirically validated taxonomy. The framework’s purpose is to organize knowledge and guide future research through unsupervised clustering analysis on multi-biomarker datasets.

## 3. Evolution of the Concept of Uremic Toxin-Driven Vascular Calcification and the Need for a Phenotypic Framework

Over the past two decades, the conceptualization of uremic toxins has undergone a remarkable transformation—from a narrow physicochemical definition to a multidimensional, translational paradigm with direct clinical implications. This evolution, while substantially advancing our mechanistic understanding, has revealed a critical gap: the need for a clinically interpretable framework that translates molecular toxicology into actionable phenotypic patterns capable of guiding risk stratification and therapeutic decision-making.

### 3.1. Early Classifications: From Biochemical Description to Mechanistic Insight

The seminal classification proposed by the European Uremic Toxin Work Group (EUTox, 2003) [17] represented the first systematic attempt to catalogue uremic solutes according to molecular weight and protein binding, dividing them into small water-soluble compounds, protein-bound solutes, and middle molecules. While this structure provided an essential biochemical and dialytic reference, it remained primarily descriptive, capturing only a fragment of the biological complexity of uremic retention solutes.

The 2021 international consensus [18] marked a pivotal step forward, introducing a mechanistic-pathway perspective that incorporated dialytic clearance efficiency, metabolic reactivity, and clinical outcome relevance. This framework acknowledged that molecular size alone does not determine toxicity; rather, the interactions between solutes and biological targets—receptors, signaling pathways, and organ tropism—define their true pathogenic potential.

### 3.2. Recent Advances: Systems Biology and Translational Taxonomy

A further evolution came with the 2023 multidimensional translational taxonomy [19], which embraced a systems-biology vision integrating omic signatures, cellular networks, and organ-specific effects. This approach connected molecular data to pathophysiological outcomes across cardiovascular, neurological, and immune systems, offering a unified structure to guide experimental and clinical nephrology alike. The integration of metabolomics, proteomics, and transcriptomics data with clinical phenotyping represented a conceptual leap toward precision nephrology, acknowledging that uremic toxicity manifests as a complex network of molecular perturbations rather than isolated biochemical derangements.

### 3.3. The Clinical Gap: From Chemical Descriptors to Phenotypic Patterns

Despite these conceptual advances, most existing classifications remain rooted in chemical descriptors, providing limited interpretability for clinicians faced with the heterogeneity of CKD phenotypes. In real-world practice, understanding uremic toxin biology is not merely a scientific exercise—it is a clinical necessity for decoding the complex interplay between molecular stressors and organ dysfunction.

Recognizing that uremic toxins exert distinct effects across multiple systems—cardiovascular, neurological, immune, and endocrine—a more clinically oriented taxonomy is required. Such a phenotypic framework would enable physicians to interpret vascular injury in the broader context of systemic uremia, allowing the identification of patient-specific profiles based on dominant biological signatures. In turn, this would facilitate therapeutic tailoring, aligning pharmacologic and depurative interventions with the individual’s molecular and vascular phenotype.

### 3.4. Toward Precision Medicine in Uremic Vascular Disease

In this light, the study of uremic toxins evolves from a cataloguing effort into a translational enterprise, aimed at linking solute accumulation to disease expression. Understanding these connections provides not only a mechanistic explanation for clinical variability but also the foundation for precision medicine in CKD-related vascular pathology. The phenotypic framework presented in the following section (Section 3) operationalizes this vision, proposing five mechanistic-clinical clusters that capture distinct biological trajectories through which uremic toxins drive vascular calcification—inflammatory-oxidative, mineral-metabolic, epigenetic-senescent, endocrine cross-talk, and integrated toxic continuum. Each phenotype represents not merely a descriptive category but a clinically actionable signature that enables biomarker-guided risk stratification, rational therapeutic selection, and personalized disease monitoring.

### 3.5. Disease Derivation of the Phenotypic Framework

The proposed classification emerged through narrative synthesis of mechanistic and clinical evidence from the uremic toxin and VC literature (2010–2024). The five phenotypes were identified through iterative analysis of recurring patterns where specific toxin clusters consistently activated convergent pathological pathways. Each phenotype was characterized by: 1—biological plausibility from experimental models, 2—clinical observability through biomarker co-occurrence in published cohorts, and 3—therapeutic relevance with differential intervention responses.

This represents a hypothesis-generating conceptual framework requiring prospective validation, not an empirically validated taxonomy. We reference narrative synthesis methodology [20] as the structured approach for integrating heterogeneous evidence, and identify unsupervised clustering analysis on multi-biomarker datasets as the critical next step for validation.

## 4. Phenotypic Clusters of Uremic Toxin-Driven Vascular Calcification

The growing recognition that uremic toxins are not merely biochemical residues but dynamic biological agents has fundamentally reshaped the understanding of VC in CKD. Beyond their physicochemical heterogeneity, these solutes activate interlaced molecular networks that drive inflammation, oxidative stress, mineral imbalance, cellular reprogramming, and endocrine disruption. To translate this molecular complexity into clinical meaning, VC can be reframed as a constellation of toxin-driven phenotypes—distinct yet interconnected biological states that capture how uremia manifests along specific vascular trajectories.

**Figure 1:**
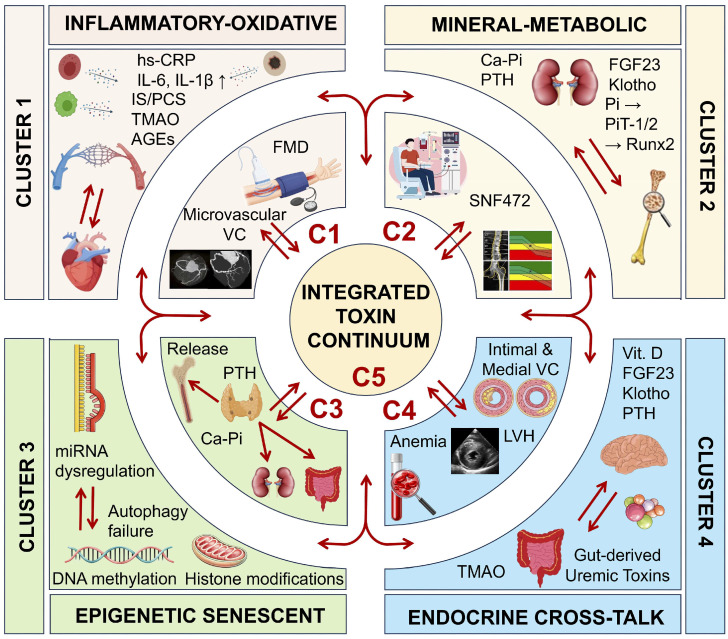
Phenotypic Classification Framework for Uremic Toxin-Driven Vascular Calcification. Arrows indicate the direction of biological effects: ↑: unidirectional; ↑↓: bidirectional; ↑↓→/↑↓←/←→↑/←→↓: multidirectional. C1: Cluster 1; C2: Cluster 2; C3: Cluster 3; C4: Cluster 4; C5: Cluster 5.

This phenotypic framework integrates molecular toxicology, systems biology, and clinical observation into a unifying vision of “uremic vascular signatures.” The five principal mechanistic-clinical clusters (Figure 1) comprise: 1—inflammatory-oxidative, 2—mineral-metabolic, 3—epigenetic-senescent, 4—endocrine cross-talk, and 5—the integrated toxic continuum—each representing a major biological route through which toxin accumulation promotes vascular transformation and accelerated cardio-renal aging (Table 1). As depicted in Figure 1, these phenotypes are interconnected through dynamic molecular cross-talk, with the integrated toxic continuum (Cluster 5: C5) representing the convergence point where all pathogenic pathways merge into a self-sustaining state of systemic vascular injury. Together, these clusters define a continuum from early adaptive responses to irreversible tissue remodeling, offering both diagnostic clarity and therapeutic direction.

### 4.1. Framework Limitations and Validation Requirements

This phenotypic classification represents a hypothesis-generating conceptual framework synthesized from convergent mechanistic and observational evidence, not an empirically validated taxonomy. The phenotypes are proposed based on patterns of toxin-pathway associations identified across experimental models and clinical cohorts, but have not been validated through prospective studies using unbiased clustering algorithms on comprehensive biomarker datasets.

Phenotypic boundaries are not absolute; overlap is expected and common in clinical practice. The framework’s purpose is organizing knowledge and guiding research, not clinical decision-making.

### 4.2. Cluster 1: The Inflammatory–Oxidative Phenotype

This phenotype manifests predominantly in maintenance hemodialysis patients with high inflammatory burden, commonly associated with protein-energy wasting, diabetes mellitus, and infectious complications [15]. The typical biomarker signature includes markedly elevated protein-bound uremic toxins (IS > 100 μM, PCS > 150 μM), high-sensitivity C-reactive protein > 10 mg/L, elevated circulating interleukin-6 (IL-6), and severe endothelial dysfunction reflected by flow-mediated dilation < 5% [21]. Patients often present with evidence of early microvascular calcification on intravascular ultrasound despite relatively preserved coronary artery calcium scores on computed tomography, suggesting that microcalcification precedes macrocalcification in this inflammatory-driven pathway.

Observational cohort studies have demonstrated that combined elevation of IS and systemic inflammatory markers predicts accelerated VC progression independently of traditional mineral metabolism parameters [12]. Post hoc analyses of large dialysis cohorts indicate that patients in the highest tertile of serum IS exhibit significantly higher cardiovascular mortality compared to the lowest tertile, even after adjustment for phosphate, calcium, and parathyroid hormone levels [22]. Similarly, data from the Japanese hemodialysis registries demonstrate that the inflammatory-oxidative phenotype—characterized by high IS/PCS and elevated CRP—is associated with younger age at first cardiovascular event and more rapid progression of pulse wave velocity, suggesting early vascular aging [23].

This phenotype represents approximately 25–35% of prevalent dialysis patients and is particularly enriched among those with inadequate dialytic clearance of middle molecules, residual renal function loss, and comorbid conditions that amplify oxidative stress [24]. Prognostically, these patients exhibit the highest responsiveness to enhanced dialysis modalities (hemodiafiltration, expanded hemodialysis) and antioxidant interventions in pilot studies, underscoring the potential for phenotype-targeted therapeutic strategies. Identification of this cluster using the biomarker panel described in Table 1 enables precision-based risk stratification and guides intensification of anti-inflammatory and dialysis optimization strategies.

### 4.3. Cluster 2: The Mineral–Metabolic Phenotype

The mineral-metabolic phenotype represents the classic presentation of CKD-mineral and bone disorder (CKD-MBD) and is the most extensively characterized VC pattern in CKD [3]. Patients typically present with advanced CKD (stages 4–5) or established dialysis dependence, characterized by persistent hyperphosphatemia (serum phosphate > 5.5 mg/dL despite phosphate binder therapy), markedly elevated intact fibroblast growth factor 23 (FGF23 > 200–500 RU/mL), secondary hyperparathyroidism (PTH > 600 pg/mL), and profound Klotho deficiency (soluble Klotho < 400 pg/mL) [24]. Imaging studies reveal predominant medial (Mönckeberg-type) arterial calcification with progressive increases in coronary artery calcium scores (typical Agatston progression 15–30% per year) and elevated pulse wave velocity reflecting arterial stiffness [5].

This phenotype accounts for an estimated 40–50% of VC cases in the dialysis population and nearly 60–70% in pre-dialysis CKD stages 4–5, making it the most prevalent pattern [1]. The EVOLVE trial and its post hoc analyses provided robust validation of this phenotype, demonstrating that patients with biochemical features of mineral-metabolic dysregulation (elevated Pi, Ca-Pi product > 55 mg^2^/dL^2^, PTH > 600 pg/mL) exhibited the most pronounced calcification burden at baseline [25]. Similarly, data from the CaLIPSO trial confirmed that the mineral-metabolic phenotype—defined by hyperphosphatemia and elevated FGF23—showed significant attenuation of coronary artery calcification progression with SNF472 treatment [25].

Clinically, this phenotype is associated with left ventricular hypertrophy, diastolic dysfunction, and increased risk of sudden cardiac death, reflecting the direct myocardial toxicity of FGF23 in addition to vascular injury [26]. Bone biopsy findings typically reveal high bone turnover (osteitis fibrosa) in the setting of severe hyperparathyroidism, although mixed uremic osteodystrophy patterns may coexist. Prognostic stratification using the biomarker panel in Table 1 identifies patients requiring intensive phosphate control (target < 5.0 mg/dL), calcimimetic therapy, and consideration for emerging crystal-growth inhibitors [27]. This phenotype serves as the primary target population for current phase 3 clinical trials of VC therapeutics and represents the most tractable subgroup for near-term pharmacological intervention.

### 4.4. Cluster 3: The Epigenetic–Senescent Phenotype

The epigenetic-senescent phenotype emerges insidiously in patients with prolonged dialysis vintage (typically >5 years) and represents a state of irreversible vascular reprogramming that persists despite normalization of traditional biochemical parameters [16]. These patients characteristically exhibit paradoxical progression of VC even under optimal phosphate control (serum phosphate 4.0–5.0 mg/dL), well-managed parathyroid hormone levels, and adequate dialysis clearance of small solutes. The biomarker signature includes persistently elevated dephosphorylated-uncarboxylated matrix Gla protein (dp-ucMGP > 1500–2000 pmol/L), markers of cellular senescence (elevated circulating p16^INK4a^-positive cells, increased senescence-associated β-galactosidase activity), and evidence of systemic oxidative damage [14].

Emerging data from long-term dialysis cohorts suggest that 15–25% of patients with dialysis vintage exceeding 5 years develop this refractory calcification pattern, with higher prevalence among those who experienced early-life uremic toxin exposure or inadequate dialysis in the initial years of renal replacement therapy [28]. Histopathological examination of arterial specimens from these patients reveals extensive elastin degradation, diffuse medial calcification with osteoid-like bone matrix formation, and accumulation of senescent cells expressing p53, p21, and senescence-associated secretory phenotype markers within the vascular wall [28]. Importantly, this phenotype exhibits minimal response to conventional CKD-MBD therapies; retrospective analyses of calcimimetic trials show that patients with long dialysis vintage and established extensive calcification derive substantially less benefit from phosphate or PTH normalization compared to incident dialysis patients.

The recognition of this phenotype has profound therapeutic implications. Standard mineral metabolism interventions, while necessary to prevent further injury, are insufficient to reverse established epigenetic-senescent reprogramming. Instead, this subgroup represents the ideal target population for emerging cellular reprogramming strategies, including sirtuin activators, senolytic agents, and autophagy-enhancing therapies currently in preclinical and early clinical development [29]. Biomarker-based identification using the panel in Table 1—particularly dp-ucMGP combined with senescence markers and long dialysis vintage—enables prognostic stratification and enrollment in trials of next-generation therapies aimed at reversing, rather than merely preventing, vascular injury. From a clinical standpoint, these patients require counseling regarding the refractory nature of their calcification and consideration for investigational cellular rejuvenation approaches [30].

### 4.5. Cluster 4: The Endocrine Cross-Talk Phenotype

The endocrine cross-talk phenotype manifests as a multisystem hormonal dysregulation syndrome extending beyond traditional CKD-mineral and bone disorder to encompass the kidney-bone-vascular axis, the gut–vascular interface, and neuroendocrine perturbations [31]. Patients typically present with complex biochemical derangements including profound vitamin D deficiency (25-hydroxyvitamin D < 20 ng/mL, 1,25-dihydroxyvitamin D undetectable), Klotho deficiency (<400 pg/mL), FGF23 elevation (>200 RU/mL) with evidence of FGF23 resistance (persistent hyperphosphatemia despite elevated FGF23), and secondary hyperparathyroidism resistant to conventional therapy [32]. Concurrently, these patients exhibit elevated gut-derived uremic toxins—particularly trimethylamine N-oxide (TMAO > 10 μM) and phenylacetylglutamine (PAGln)—reflecting intestinal dysbiosis with depletion of beneficial short-chain fatty acid-producing bacteria and expansion of proteolytic species [33]. Clinical characteristics of this phenotype include not only VC but also broader manifestations of hormonal imbalance. These include refractory anemia [34] despite adequate iron stores and erythropoiesis-stimulating agent therapy (linked to hepcidin dysregulation and FGF23-mediated erythropoietin resistance) [35,36,37], severe pruritus (associated with imbalanced bile acid metabolism), and cognitive dysfunction (related to uremic encephalopathy and gut–brain axis disruption) [23].

Cardiovascular imaging reveals a mixed pattern of vascular injury, with both atherosclerotic (intimal) and medial calcification, endothelial dysfunction, and increased arterial stiffness. Importantly, bone histomorphometry in these patients often demonstrates adynamic bone disease or mixed uremic osteodystrophy rather than pure high-turnover patterns, reflecting the complex interplay between PTH resistance, vitamin D deficiency, and FGF23 excess.

Cross-sectional studies and post hoc analyses of vitamin D supplementation trials suggest that a substantial proportion of CKD stage 4–5 and dialysis patients exhibit features consistent with this phenotype, with enrichment among those with long-standing diabetes, polypharmacy, and proton pump inhibitor use (which further disrupts calcium absorption and gut microbiome) [27,37].

This subgroup appears uniquely positioned to benefit from selective vitamin D receptor activators [37], microbiome-targeted interventions (prebiotics, probiotics, dietary fiber), and iron supplementation strategies aimed at reducing FGF23 production [36]. Identification using the biomarker panel in Table 1—particularly combined assessment of Klotho, FGF23, vitamin D status, and gut-derived metabolites—enables recognition of this complex endocrine phenotype and guides multidimensional therapeutic approaches targeting the kidney–bone–gut–vascular axis.

### 4.6. Cluster 5: The Integrated Toxic Continuum

The integrated toxic continuum represents the convergence point of advanced CKD where multiple pathogenic pathways—inflammatory, metabolic, epigenetic, and endocrine—merge into a self-sustaining state of systemic toxicity [15]. Clinically, these patients present with end-stage or advanced CKD (stage 5 or 5D) characterized by simultaneous derangements across all biomarker domains. These include severe hyperphosphatemia (>6.0 mg/dL), markedly elevated FGF23 (>500–1000 RU/mL), profound Klotho deficiency, PTH > 800–1000 pg/mL, high IS (>150 μM) and PCS (>200 μM), systemic inflammation (hs-CRP >10 mg/L), and extensive VC burden (coronary artery calcium Agatston score > 400, often >1000) [1]. Cardiovascular imaging reveals diffuse atherosclerotic and medial calcification affecting coronary arteries, aorta, and peripheral vessels, accompanied by severe arterial stiffness (pulse wave velocity > 12–15 m/s), left ventricular hypertrophy, valvular calcification (particularly aortic valve) [8], and diastolic dysfunction.

This phenotype is not merely an additive combination of individual cluster features but represents a qualitatively distinct state in which the organism itself becomes the source of ongoing vascular injury. The vascular wall transitions from a passive target to an active endocrine organ secreting inflammatory mediators (IL-6, TNF-α), calcifying extracellular vesicles, senescence-associated secretory phenotype factors, and procoagulant molecules that propagate injury systemically [37]. Multi-organ manifestations are characteristic: cardiovascular (heart failure, arrhythmias, sudden cardiac death), neurological (uremic encephalopathy, cognitive decline), hematologic (refractory anemia, platelet dysfunction), immunologic (chronic inflammation, infection susceptibility), and musculoskeletal (fracture risk from renal osteodystrophy and vascular steal). Critically, these patients exhibit minimal or paradoxical responses to single-pathway interventions; normalization of phosphate alone, PTH suppression, or inflammation reduction each fail to substantially alter disease trajectory because the pathogenic network has acquired self-reinforcing properties [16].

The integrated toxic continuum accounts for an estimated 15–25% of prevalent dialysis patients and is strongly associated with dialysis vintage > 3 years, multiple comorbidities (diabetes, heart failure, prior cardiovascular events), and inadequate early-stage CKD management [38]. Prognostically, this phenotype carries the highest cardiovascular mortality risk, with 5-year survival rates of 30–40% in most cohorts—substantially lower than other phenotypic patterns. Data from large registry studies and clinical trials consistently demonstrate that patients with features of integrated continuum (defined by simultaneous elevation of ≥4 biomarker domains from Table 1) exhibit the poorest outcomes and are least responsive to monotherapy approaches [39].

Recognition of this phenotype has critical therapeutic and prognostic implications. These patients require multi-dimensional combination therapy addressing oxidative stress, inflammation, mineral metabolism, and endocrine dysfunction simultaneously, as exemplified by protocols combining intensive dialysis, Pi binders, calcimimetics, vitamin D receptor activators, antioxidants, and iron supplementation [40]. Despite maximal medical therapy, however, disease modification remains limited, underscoring the importance of early intervention before transition to the integrated continuum occurs. From a clinical trial perspective, this population represents both the highest unmet medical need and the most challenging therapeutic target; future studies must acknowledge phenotypic heterogeneity and avoid enrollment of predominantly end-stage continuum patients in trials of single-pathway interventions likely to show minimal efficacy [41,42]. Ultimately, the integrated toxic continuum defines the “point of no return” in uremic vascular pathology—a state where prevention has failed and restoration of vascular integrity may require transformative approaches such as cellular reprogramming, regenerative medicine, or renal replacement that fully restores kidney function (successful transplantation).

Recognition of this phenotype using Table 1 biomarkers enables honest prognostic discussions, intensification of palliative and supportive care, and prioritization for kidney transplantation when feasible. While the integrated continuum represents the most refractory and clinically challenging endpoint of uremic vascular injury, the broader phenotypic framework provides opportunities for earlier intervention and phenotype- targeted therapeutic selection across the full disease spectrum.

The five mechanistic-clinical clusters described above (Figure 1) collectively define the phenotypic spectrum of uremic toxin-driven VC in CKD. While each cluster exhibits distinct dominant features—inflammatory-oxidative (Cluster 1: C1), mineral-metabolic (Cluster 2: C2), epigenetic-senescent (Cluster 3: C3), endocrine cross-talk (Cluster 4: C4), or integrated continuum (Cluster 5: C5)—these phenotypes are not mutually exclusive. Rather, they represent biological states along a dynamic continuum, with patients potentially transitioning between phenotypes as disease progresses or exhibiting overlapping characteristics, particularly in advanced stages. The recognition that the integrated toxic continuum C5 serves as the convergence point for all pathogenic pathways underscores the importance of early phenotypic identification and targeted intervention before transition to this refractory state occurs. This phenotypic framework, supported by the comprehensive biomarker panel (Table 2), enables precision-based risk stratification and guides individualized therapeutic selection, as detailed in subsequent sections.

### 4.7. Managing Mixed Phenotypes

In clinical practice, many patients present with biomarker elevations spanning multiple phenotypic domains, reflecting the complex and overlapping nature of uremic toxin-driven vascular injury. For instance, a patient may exhibit simultaneously elevated FGF23 consistent with mineral-metabolic dysregulation alongside markedly increased indoxyl sulfate and high-sensitivity CRP indicating concurrent inflammatory-oxidative stress. Such mixed phenotypic presentations require a hierarchical therapeutic approach that prioritizes interventions targeting the dominant phenotype—identified by the highest magnitude of biomarker elevation or the most clinically significant pathway—while maintaining surveillance of secondary features through serial biomarker monitoring.

Sequential management becomes particularly important in patients with transitioning phenotypes, where the mechanistic profile evolves over time. A patient initially characterized by isolated mineral-metabolic disturbances may progressively develop inflammatory and epigenetic features as uremic exposure persists, ultimately evolving toward the integrated toxic continuum phenotype. In such cases, therapeutic strategy must be dynamically adjusted in response to shifting biomarker profiles, typically reassessed at 3- to 6-month intervals.

As a practical illustration, consider a hemodialysis patient presenting with moderately elevated FGF23 (800 pg/mL) and markedly elevated indoxyl sulfate (150 μM) plus high-sensitivity CRP (15 mg/L). The therapeutic priority should focus on anti-inflammatory interventions—intensified dialysis modality or oral adsorbents to reduce toxin burden—alongside standard phosphate binder optimization. Serial biomarker reassessment would guide treatment modification based on the evolving phenotypic predominance.

#### Clinical Feasibility and Biomarker Standardization

The proposed biomarker panel encompasses markers with varying clinical availability and analytical standardization. Routine implementation relies on widely available biomarkers with established assay standardization: serum phosphate, calcium, intact parathyroid hormone, fibroblast growth factor 23, 25-hydroxyvitamin D, and high-sensitivity C-reactive protein. These are measurable in most clinical laboratories using standardized methodologies with well-characterized reference ranges.

A second tier includes indoxyl sulfate, p-cresyl sulfate, and dephosphorylated-uncarboxylated matrix Gla protein, requiring specialized analytical platforms typically available only in referral centers or research laboratories. For these protein-bound uremic toxins and vitamin K-dependent proteins, significant inter-assay variability persists due to absent internationally harmonized reference standards. The European Uremic Toxin Work Group has advanced standardization of indoxyl sulfate and p-cresyl sulfate measurement through reference methods based on liquid chromatography-tandem mass spectrometry. The International Federation of Clinical Chemistry and Laboratory Medicine similarly aims to harmonize fibroblast growth factor 23 assays, which currently exhibit considerable variability between intact and C-terminal measurement approaches.

A third category encompasses research-only markers: circulating senescent cell populations, extracellular vesicle composition, and senescence-associated secretory phenotype factors. These lacks validated clinical assays and standardized protocols, limiting applicability to investigational studies.

For clinical implementation, we recommend a tiered approach. Routine phenotypic assessment employs the core panel of widely available biomarkers. Specialized markers remain reserved for research protocols or phenotype-enriched clinical trials. As analytical methodologies mature and international standardization initiatives advance, the biomarker panel can progressively incorporate current investigational markers demonstrating clinical utility and analytical reliability.

## 5. Promoters and Inhibitors of Vascular Calcification

**Figure 2:**
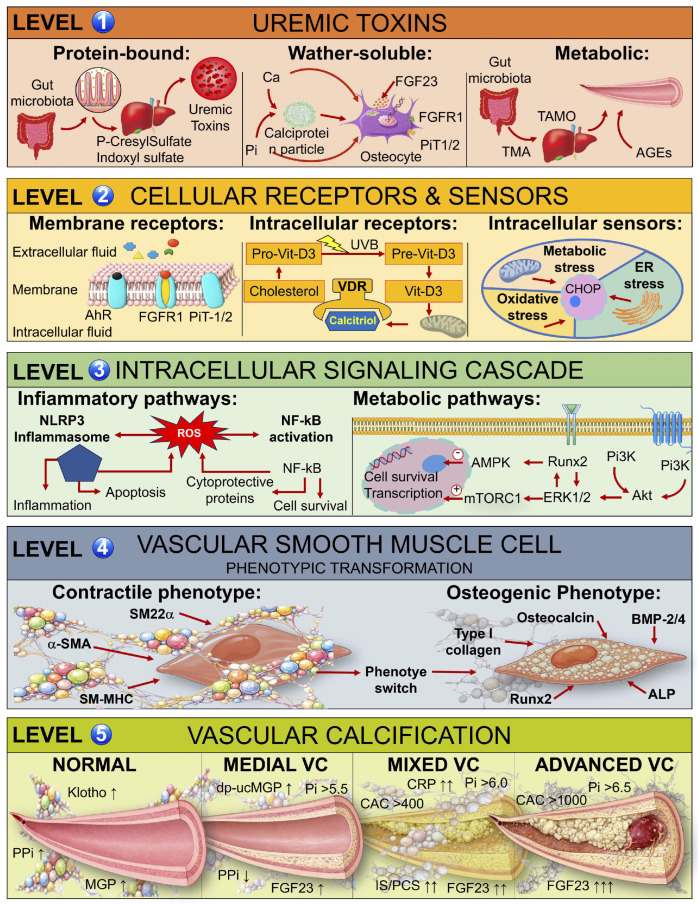
Mechanisms of VSMC transformation leading to Vascular Calcification. ↓: decrease; ↑: increase mild; ↑↑: increasemoderate; ↑↑↑: increase marked, indicate greater intensity.

VC represents one of the most emblematic expressions of CKD-related vascular injury. Far from being an inert mineral deposition, VC is now recognized as an actively regulated, cell-mediated process that mirrors physiological bone formation. In CKD, this transformation results from the convergence of metabolic, hormonal, inflammatory, and molecular disturbances that reprogram VSMCs, endothelial cells, and macrophages toward an osteo-chondrogenic fate. The lesions that emerge—predominantly within the medial layer—lead to progressive arterial stiffness, left ventricular hypertrophy, and a sharp increase in cardiovascular mortality. Within the multidimensional framework established earlier, these mechanisms constitute the molecular substrate of the five phenotypic clusters described above: inflammatory-oxidative, mineral-metabolic, epigenetic-senescent, endocrine cross-talk, and integrated toxic continuum. VC can therefore be conceptualized as the net result of an imbalance between pro-calcific drivers (promoters) and anti-calcific defenses (inhibitors), whose interplay determines whether the vascular wall follows a degenerative or adaptive trajectory. Importantly, the relative dominance of specific promoters and the failure of particular inhibitory mechanisms define which phenotypic pattern predominates in individual patients, thereby informing precision- targeted therapeutic strategies. The molecular cascade linking uremic toxin accumulation to VC proceeds through five sequential mechanistic levels (Figure 2): 1—uremic toxin generation and accumulation (protein-bound, water-soluble, and metabolic toxins), 2—engagement of cellular receptors and stress sensors (membrane receptors, intracellular vitamin D receptor, oxidative and metabolic stress sensors), 3—activation of intracellular signaling cascades (inflammatory and metabolic pathways), 4—vascular smooth muscle cell phenotypic transformation (contractile to osteogenic switch), and 5—progressive VC (from normal vessel architecture through medial, mixed, and advanced calcification with thrombotic complications). This five-level framework integrates the promoters and inhibitors of calcification into a unified pathogenic model, providing mechanistic insight into how each phenotypic cluster emerges from distinct patterns of molecular dysregulation.

### 5.1. Promoters of Vascular Calcification

#### 5.1.1. Mineral and Metabolic Imbalance

Among the earliest and most potent drivers of VC is hyperphosphatemia, a hallmark of CKD-mineral and bone disorder (CKD-MBD). Elevated extracellular phosphate (Pi) promotes supersaturation and precipitation of Ca-Pi complexes, initiating mineral nucleation within the vascular extracellular matrix [3]. Phosphate enters VSMCs via sodium-dependent transporters PiT-1 and PiT-2 [24,43,44,45], activating ERK1/2, NF-κB, and Smad signaling pathways. These cascades converge on Runx2, the master transcriptional regulator of osteoblastic differentiation, which induces ALP, osteocalcin, and type I collagen while repressing contractile markers such as SM22α and smooth muscle myosin heavy chain (SM-MHC) [46,47,48]. Upstream mediators, notably Msx2 and bone morphogenetic proteins (BMP-2/4), amplify Runx2 activation, integrating phosphate toxicity with oxidative and mechanical stress. The downstream transcription factor Osterix (Sp7) consolidates this osteogenic phenotype, promoting hydroxyapatite formation [49,50]. Calcium excess acts synergistically, stabilizing amorphous Ca-Pi clusters and accelerating crystallization within apoptotic bodies and extracellular vesicles (EVs) that contain annexins, ALP, and phospholipids [40,46]. Thus, mineral imbalance does not merely trigger passive precipitation but initiates a biologically orchestrated transdifferentiation of the vascular wall.

This phosphate-driven pathway constitutes the primary mechanism underlying the mineral-metabolic phenotype, where persistent hyperphosphatemia, elevated FGF23, and Klotho deficiency drive VSMC osteogenic transformation. Patients presenting with this phenotype exhibit predominant medial calcification with rapid Agatston score progression—typically 15–30% per year—and represent the primary candidates for intensive phosphate control, calcimimetics, and emerging therapies targeting the Pi-FGF23-Klotho axis [23,47,48].

At the systemic level, Pi retention coexists with hypocalcemia, reduced calcitriol synthesis, and Klotho deficiency, all of which drive secondary hyperparathyroidism (sHPT). Initially adaptive, chronic PTH excess becomes maladaptive as it mobilizes Ca and Pi from bone, exacerbating vascular exposure to calcifying substrates. In VSMCs, PTH enhances intracellular Ca^2+^ influx through L-type channels and activates the PKC-MAPK axis, increasing Runx2, ALP, and osteocalcin transcription [24]. The result is a hormonal-metabolic loop that reinforces the osteogenic switch at both systemic and cellular levels. This maladaptive PTH-driven pathway is particularly evident in the endocrine cross-talk phenotype [32], where profound vitamin D deficiency and PTH resistance coexist with mineral imbalance, creating a complex multisystem endocrine syndrome [31,49].

#### 5.1.2. Hormonal and Regulatory Factors

The FGF23-Klotho axis plays a pivotal role in mineral homeostasis and vascular pathology. Under physiological conditions, fibroblast growth factor 23 (FGF23) acts through Klotho-dependent signaling to enhance phosphate excretion [3] and suppress calcitriol synthesis. In CKD, however, Klotho deficiency disrupts this equilibrium, converting FGF23 signaling into a maladaptive cascade. Unchecked activation of FGFR1c/3c in vascular and cardiac tissues [31] induces ERK1/2 and PLCγ pathways, promoting oxidative stress, ALP activity, and suppression of local Klotho and Fetuin-A [31,49]. The result is a pathological FGF23 gain of function that promotes endothelial dysfunction and vascular mineralization [10]. Emerging evidence has linked FGF23 toxicity to iron deficiency and hypoxia signaling [50]. Low iron stabilizes HIF-1α [51], upregulating FGF23 transcription and cleavage via furin, producing biologically active C-terminal fragments [32,52]. This iron-FGF23-hypoxia axis forms a vicious circle in which iron deficiency amplifies oxidative stress, suppresses erythropoiesis, and further elevates FGF23 levels. Conversely, oral iron repletion may attenuate FGF23 hypersecretion, providing a metabolic strategy to rebalance the phosphate-FGF23-Klotho triad [53].

FGF23-Klotho dysregulation represents the hallmark feature of both the mineral-metabolic and endocrine cross-talk phenotypes. In the mineral-metabolic phenotype, markedly elevated FGF23 (>200–500 RU/mL) with profound Klotho deficiency drives direct vascular toxicity alongside phosphate retention, making patients in this cluster prime candidates for emerging FGF23-targeted therapies and ENPP1 enzyme replacement strategies. In the endocrine cross-talk phenotype, this hormonal imbalance is compounded by gut dysbiosis, vitamin D deficiency, and PTH resistance, creating a multisystem endocrine syndrome that requires integrated hormonal and microbiome-targeted therapy rather than isolated mineral metabolism correction [31,37,49,54].

Vitamin D adds another layer of complexity to the hormonal landscape of uremic vascular injury. Adequate 1,25(OH)_2_-vitamin D maintains Ca-Pi equilibrium, limits PTH hypersecretion, and exerts anti-inflammatory effects. In CKD, reduced 1α-hydroxylase activity and urinary protein loss lead to vitamin D deficiency, contributing to sHPT, RAAS activation, and endothelial dysfunction [52]. Yet excessive VDR activation—especially under hypercalcemic or hyperphosphatemic conditions—may paradoxically induce osteogenic differentiation by upregulating Runx2 and ALP [31]. This biphasic behavior underscores the need for a nuanced therapeutic approach: maintaining sufficient vitamin D levels to prevent sHPT while avoiding overactivation of VDR signaling in calcification-prone states. This delicate balance is particularly critical in patients presenting with either the mineral-metabolic or endocrine cross-talk phenotypes, where vitamin D receptor activation must be carefully titrated to avoid exacerbating pre-existing mineral dysregulation while still achieving PTH suppression and systemic anti-inflammatory benefits.

#### 5.1.3. Inflammation, Oxidative Stress, and Endothelial Dysfunction

Chronic inflammation and oxidative stress represent key amplifiers of VC in CKD. The uremic milieu—rich in protein-bound toxins such as indoxyl sulfate (IS) and p-cresyl sulfate (PCS)—activates NF-κB signaling in endothelial cells and macrophages, driving cytokine release (IL-1β, IL-6, TNF-α) and ROS production [24]. These cytokines induce NLRP3 inflammasome activation and osteogenic gene expression in VSMCs [55]. Concurrently, IS and PCS stimulate the aryl hydrocarbon receptor (AhR)/p38 MAPK pathway, enhancing apoptosis and vesicle-mediated mineralization [28]. At the endothelial interface, NO depletion and oxidative imbalance promote endothelial-to-mesenchymal transition (EndMT), wherein endothelial cells acquire mesenchymal and osteogenic features. Senescent endothelium releases microvesicles enriched in annexins, ALP, and BMP-2 that propagate calcific signals to VSMCs [56]. Mechanical stressors—hypertension, disturbed flow, and arterial stiffness—exacerbate these processes via TGF-β/Smad and MAPK activation [57]. Thus, inflammatory and hemodynamic forces act synergistically, transforming the endothelium from a protective barrier into an initiator of calcification.

This inflammatory-oxidative cascade defines the phenotypic signature of patients characterized by markedly elevated IS (>100 μM) and PCS (>150 μM), high-sensitivity CRP exceeding 10 mg/L, and severe endothelial dysfunction reflected by flow-mediated dilation below 5%. These patients—typically on maintenance hemodialysis with inadequate middle-molecule clearance, concurrent diabetes, or recurrent infectious complications—exhibit early microvascular calcification detectable on intravascular ultrasound that often precedes macrocalcification visible on CT imaging. This inflammatory-oxidative phenotype represents the primary target population for intensified dialysis modalities such as hemodiafiltration and expanded hemodialysis, oral toxin adsorbents like AST-120, antioxidant therapies, and emerging NLRP3 inflammasome inhibitors currently in early-phase clinical development [12,21,22,23,24,37].

The role of EndMT in perpetuating vascular injury deserves particular emphasis in this context. IS and PCS drive endothelial cells toward a pro-calcific mesenchymal state through chromatin remodeling and microRNA dysregulation, particularly suppression of miR-30c and other microRNAs that normally maintain endothelial identity [14]. This mechanism is acutely prominent in the inflammatory-oxidative phenotype, where it can potentially be reversed through toxin removal and antioxidant intervention. However, it also contributes to the irreversible vascular reprogramming observed in the epigenetic-senescent phenotype. In long-term dialysis patients with vintage exceeding five years, persistent uremic toxin exposure induces stable epigenetic changes (DNA methylation, histone modifications, and durable microRNA dysregulation). These changes perpetuate vascular injury despite normalization of biochemical parameters, creating a refractory calcification pattern [27,28,29,30].

#### 5.1.4. Intracellular Stress, Mitochondria, and Autophagy Deficiency

VSMCs in CKD exhibit profound intracellular derangements that extend beyond surface receptor signaling to fundamental disruptions in cellular homeostasis. Mitochondrial dysfunction leads to membrane depolarization, ATP depletion, and mtDNA damage, increasing reactive oxygen species production and triggering senescence through the p53/p21 axis. Simultaneously, endoplasmic reticulum (ER) stress—mediated by the PERK-eIF2α-ATF4-CHOP pathway—induces apoptosis and enhances Runx2 transcription [38,58]. When autophagy is impaired, damaged mitochondria and calcifying vesicles accumulate, amplifying oxidative stress and mineral deposition. Restoration of autophagy via AMPK-mTORC1 modulation has been shown experimentally to reverse these changes and restore the contractile phenotype, highlighting the therapeutic potential of autophagy-enhancing interventions.

While mitochondrial dysfunction and autophagy impairment are universal features across all phenotypic clusters, their predominance and reversibility vary substantially according to phenotypic context. In the epigenetic-senescent phenotype observed in long-term dialysis patients, irreversible mitochondrial damage and persistent autophagy failure—driven by epigenetic silencing of autophagy genes such as ATG5, ATG7, and BECN1—result in cellular senescence and activation of the senescence-associated secretory phenotype (SASP). This SASP state perpetuates inflammation and calcification even after correction of upstream metabolic triggers, creating a self-sustaining cycle of vascular injury that conventional autophagy activators cannot reverse, necessitating instead senolytic therapies or cellular reprogramming approaches [33,44]. In contrast, in the inflammatory-oxidative phenotype, IS and PCS directly impair mitochondrial function and suppress autophagic flux through AMPK inhibition and mTORC1 hyperactivation, creating a feedforward cycle of oxidative injury that may be partially reversible through toxin removal, enhanced dialysis clearance, and pharmacologic AMPK activation [28,37,39,55]. In the mineral-metabolic phenotype, phosphate toxicity impairs autophagy through ER stress pathways, suggesting that combined phosphate control and autophagy-enhancing strategies—such as ferric citrate (which both binds phosphate and stimulates autophagy) [59], metformin (AMPK activator), or SGLT2 inhibitors (which activate AMPK and enhance mitophagy)—may provide synergistic vascular protection [60,61,62].

#### 5.1.5. Extracellular Vesicles, Calciprotein Particles, and Immune Cross-Talk

Extracellular vesicles (EVs) and calciprotein particles (CPPs) act as physical vectors translating cellular stress into extracellular mineralization, bridging the gap between intracellular metabolic derangements and tissue-level calcification. Under normal conditions, inhibitors such as Fetuin-A, matrix Gla protein (MGP), and inorganic pyrophosphate (PPi) stabilize nascent Ca-Pi complexes in a soluble, non-pathogenic form [37]. In CKD, progressive depletion of these inhibitors permits maturation of initially amorphous calciprotein particles (Type I CPPs) into dense, crystalline Type II CPPs with potent pro-inflammatory properties. These Type II CPPs activate Toll-like receptors (TLR2/4) on macrophages and endothelial cells, stimulating IL-6 and TNF-α release and reinforcing systemic inflammation, which in turn promotes further VSMC apoptosis, EV release, and CPP formation—a self-amplifying pathological circuit [38,58].

The immune system contributes directly to this vicious cycle through complex vascular-immune cross-talk [54]. M1 macrophages, polarized toward a pro-inflammatory phenotype by uremic toxins and CPPs, release cytokines that promote osteogenic signaling in VSMCs, whereas M2 macrophages exert partial protective effects through IL-10 and TGF-β secretion, though their anti-calcific activity is often insufficient to counterbalance the pro-inflammatory milieu [53]. Th17 cells produce IL-17A, which induces endothelial senescence and fibroblast proliferation, further contributing to vascular remodeling [63]. This chronic immune-vascular cross-talk establishes a self-perpetuating inflammatory-calcific circuit that becomes central to CKD progression and refractory to single-pathway therapeutic intervention.

The EV/CPP-mediated pathway and immune dysregulation exhibit phenotype-specific patterns of activity and therapeutic implications. In the inflammatory-oxidative phenotype, high circulating levels of IS and PCS drive massive VSMC apoptosis and EV release, creating a pro-calcific milieu where circulating EVs enriched in annexins and ALP serve as both disease biomarkers and potential therapeutic targets. Strategies aimed at reducing toxin burden through intensified dialysis, oral adsorbents, or emerging AhR antagonists may interrupt this EV-driven calcification cascade in patients with this phenotypic profile [12,37,39]. Conversely, in the integrated toxic continuum—representing the convergence of inflammation, oxidative stress, mineral dysregulation, and inhibitor depletion in advanced disease—maximal CPP formation, profound Fetuin-A depletion, and unrestrained immune activation create a state of uncontrolled vascular injury where single-pathway interventions targeting only one aspect of the pathogenic network inevitably fail. This phenotype requires multi-dimensional combination therapy addressing inflammation, mineral metabolism, and inhibitor repletion simultaneously [16,38]. The prominence of M1 macrophage polarization and IL-17A-mediated vascular injury in the inflammatory-oxidative phenotype additionally suggests potential benefit from targeted immunomodulatory therapies—NLRP3 inflammasome inhibitors, IL-6 antagonists, or Th17-targeting biologics—in carefully selected patient populations with biomarker-confirmed inflammatory dominance [14,53].

### 5.2. Physiological Inhibitors of Vascular Calcification

Despite the multitude of pro-calcific insults imposed by the uremic milieu, the vasculature maintains a robust network of endogenous inhibitors that preserve mineral homeostasis and structural integrity under physiological conditions. The decline or dysfunction of these defense systems constitutes a crucial determinant of calcification susceptibility in CKD. Critically, the specific pattern of inhibitor depletion—whether predominantly affecting circulating factors (Fetuin-A, MGP), enzymatic systems (ENPP1, PPi generation), or cellular mechanisms (autophagy, Klotho signaling)—helps define phenotypic vulnerabilities and informs precision-targeted therapeutic strategies aimed at restoring these protective pathways.

#### 5.2.1. Circulating and Mineral Inhibitors

Fetuin-A, a hepatic glycoprotein also known as α2-Heremans-Schmid glycoprotein, forms soluble complexes with nascent Ca-Pi aggregates (primary calciprotein particles, CPP-I), preventing their maturation into crystalline deposits and subsequent vascular incorporation. Low Fetuin-A levels, frequent in dialysis patients and those with chronic inflammatory states, correlate inversely with VC severity and cardiovascular mortality in numerous observational cohorts [24,28]. Fetuin-A deficiency is particularly pronounced in patients presenting with the inflammatory-oxidative phenotype, where chronic systemic inflammation suppresses hepatic Fetuin-A synthesis, and in the integrated toxic continuum, where multiple converging stressors—inflammation, oxidative damage, metabolic derangement, and hepatic dysfunction—synergistically deplete circulating inhibitor reserves [16,37].

Matrix Gla protein (MGP), a vitamin K-dependent peptide synthesized locally by VSMCs and endothelial cells, functions as one of the most potent endogenous inhibitors of soft tissue calcification. MGP directly binds and inactivates BMP-2, preventing its osteogenic signaling, and also interferes with hydroxyapatite crystal nucleation within the vascular matrix [43]. However, MGP requires post-translational γ-carboxylation by vitamin K-dependent γ-glutamyl carboxylase to achieve biological activity. The inactive, dephosphorylated-uncarboxylated form of MGP (dp-ucMGP) accumulates in vitamin K-deficient states and serves as both a biomarker of deficiency and a predictor of VC progression and cardiovascular mortality.

Elevated dp-ucMGP levels exceeding 1500–2000 pmol/L are the defining biomarker of the epigenetic-senescent phenotype. In this phenotype, the elevation reflects not only dietary vitamin K deficiency but also persistent epigenetic silencing of the MGP carboxylation machinery—specifically, DNA hypermethylation at the GGCX (γ-glutamyl carboxylase) gene promoter and histone deacetylation at MGP regulatory regions. This epigenetic dysregulation renders the pathway refractory to vitamin K_2_ supplementation despite normalization of circulating vitamin K levels [14,27,28,29,30]. Dp-ucMGP is also elevated in the endocrine cross-talk phenotype, where intestinal dysbiosis, malabsorption syndromes, proton pump inhibitor use, and complex vitamin D-K metabolic interactions impair vitamin K absorption and utilization, identifying patients who may benefit from aggressive vitamin K_2_ (menaquinone-7) supplementation coupled with gut microbiome optimization [31,33,64,65,66].

Gla-rich protein (GRP) acts synergistically with MGP to stabilize the vascular extracellular matrix and prevent mineralization, while osteopontin (OPN) and osteoprotegerin (OPG) bind directly to hydroxyapatite crystals and suppress RANKL-mediated osteoclastic and osteogenic signaling cascades [21,67]. Among inorganic inhibitors, inorganic pyrophosphate (PPi) stands as the most potent physiological defense against pathological mineralization. PPi is generated through ENPP1-mediated hydrolysis of extracellular ATP and actively binds Ca^2+^ ions at crystal nucleation sites, preventing both initial mineral nucleation and subsequent crystal propagation. In CKD, a catastrophic imbalance develops: decreased expression of PPi-generating enzymes (ENPP1, ANK, ABCC6) combined with increased activity of tissue-nonspecific alkaline phosphatase (TNAP), which degrades PPi to inorganic phosphate, drastically reduces vascular PPi availability and tips the balance decisively toward mineralization [43]. This severe PPi depletion coupled with elevated TNAP activity represents a hallmark biochemical signature of the mineral-metabolic phenotype, where phosphate overload and hormonal dysregulation (FGF23 excess, Klotho deficiency, PTH elevation) amplify the PPi/TNAP imbalance and create ideal conditions for hydroxyapatite crystal formation. This pathophysiological pattern identifies the mineral-metabolic phenotype as the optimal target population for emerging TNAP inhibitors and ENPP1 enzyme replacement therapy (INZ-701), both of which aim to restore the PPi/Pi balance and recreate a vascular microenvironment hostile to mineral deposition [50,68,69,70,71].

Magnesium (Mg^2+^) exerts multiple anti-calcific effects: it competes with Ca^2+^ at nucleation sites, stabilizes amorphous Ca-Pi phases and prevents their transformation into crystalline hydroxyapatite, and modulates cellular signaling by counteracting Wnt/β-catenin activation and promoting endothelial nitric oxide synthesis [3,55]. Citrate similarly chelates ionized calcium and limits crystal aggregation, serving as a local buffer within the vascular microenvironment. While magnesium and citrate depletion can occur across all phenotypic patterns, they are particularly critical in the mineral-metabolic and integrated toxic continuum phenotypes, where mineral supersaturation is maximal and every available inhibitory mechanism must be preserved or therapeutically augmented to mitigate relentless calcification progression.

#### 5.2.2. Cellular and Endogenous Mechanisms

At the cellular level, autophagy serves as a critical homeostatic mechanism that preserves the contractile phenotype of VSMCs. It facilitates clearance of damaged organelles, protein aggregates, and pro-calcific extracellular vesicles before they can seed mineral deposition. Activation of autophagy—via AMPK stimulation or mTORC1 inhibition—prevents accumulation of calcification-prone cellular debris and maintains VSMC plasticity [38]. Conversely, impaired autophagy leads to intracellular accumulation of dysfunctional mitochondria, apoptotic bodies, and mineral-laden vesicles, creating intracellular nucleation sites that ultimately manifest as tissue-level calcification [58].

The drivers and reversibility of autophagy dysfunction exhibit marked phenotype-specificity with direct therapeutic implications. In the inflammatory-oxidative phenotype, IS and PCS directly suppress autophagic flux through AMPK inhibition and mTORC1 hyperactivation, creating a potentially reversible impairment that may respond to toxin removal via intensified dialysis and pharmacologic AMPK activation with agents such as metformin or SGLT2 inhibitors [37,39]. In the epigenetic-senescent phenotype, persistent epigenetic silencing of core autophagy genes (ATG5, ATG7, BECN1) creates an irreversible autophagy-deficient state. Conventional AMPK activators cannot overcome this impairment, necessitating instead senolytic therapies (dasatinib-quercetin) or cellular reprogramming approaches aimed at reversing the underlying epigenetic landscape [28,29,30]. In the mineral-metabolic phenotype, phosphate toxicity impairs autophagy through endoplasmic reticulum stress pathways, suggesting that combined strategies addressing both phosphate burden and autophagy restoration—such as ferric citrate (phosphate binding plus autophagy stimulation), metformin, or SGLT2 inhibitors—may provide synergistic vascular protection [59,60,61].

Klotho is a transmembrane protein primarily expressed in renal tubular cells and released in soluble form into the circulation. It exerts pleiotropic systemic anti-calcific effects that extend far beyond its canonical role as the FGF23 co-receptor. Klotho inhibits sodium-dependent phosphate transporters PiT-1 and PiT-2 in VSMCs, directly reducing cellular phosphate uptake; suppresses Wnt/β-catenin signaling, thereby preventing osteogenic reprogramming; reduces oxidative stress through upregulation of antioxidant enzymes; and modulates calcium channel activity [32]. Klotho deficiency, defined as soluble Klotho levels below 400 pg/mL, represents a unifying biochemical feature of both the mineral-metabolic and endocrine cross-talk phenotypes, functioning simultaneously as a disease biomarker, a pathogenic mediator, and a rational therapeutic target. Klotho supplementation via recombinant protein administration or pharmacologic activation via small-molecule Klotho inducers represents a precision intervention strategy particularly suited for these phenotypes [72]. Similarly, bone morphogenetic protein-7 (BMP7) counteracts the pro-calcific effects of BMP-2 and BMP-4, promoting endothelial repair and extracellular matrix remodeling while suppressing Runx2-mediated osteogenic differentiation [21,32].

Endogenous antioxidant systems—including superoxide dismutase (SOD), catalase, glutathione peroxidase, and the thioredoxin system—neutralize reactive oxygen species and limit NF-κB activation, thereby interrupting oxidative stress-driven calcification cascades. Sirtuins (SIRT1, SIRT3), NAD^+^-dependent deacetylases, maintain mitochondrial integrity, restrain cellular senescence through deacetylation of p53 and other senescence mediators, reduce inflammatory signaling, and promote autophagy [28]. Together, these cellular defense systems define a molecular shield that preserves vascular elasticity and contractile phenotype. Antioxidant enzyme depletion and sirtuin downregulation are particularly severe in the inflammatory-oxidative phenotype, where uremic toxins directly suppress antioxidant defenses and overwhelm ROS scavenging capacity, and in the epigenetic-senescent phenotype, where age-related and uremia-accelerated NAD^+^ depletion impairs sirtuin activity. Therapeutic strategies targeting sirtuin activation through NAD^+^ precursor supplementation (nicotinamide riboside, nicotinamide mononucleotide) and polyphenolic sirtuin activators (resveratrol) alongside direct antioxidant supplementation show particular mechanistic promise in these phenotypic contexts [29,73,74].

#### 5.2.3. Epigenetic and Molecular Regulators

Epigenetic regulation represents an additional layer of vascular defense, wherein non-coding RNAs and chromatin modifications modulate the balance between contractile and osteogenic gene programs in VSMCs. MicroRNAs such as miR-30b, miR-143/145, and miR-204 suppress expression of Runx2 and BMP2, maintaining smooth muscle cell identity and preventing osteochondrogenic transdifferentiation [27]. Long non-coding RNAs (lncRNAs) exert dual regulatory roles: protective lncRNAs such as ANCR and GAS5 inhibit osteogenic gene transcription and promote autophagy, preserving vascular homeostasis, whereas pro-calcific lncRNAs such as H19 and SENCR facilitate endoplasmic reticulum stress responses and osteogenic differentiation. The balance between these opposing regulatory RNAs delineates individual susceptibility to VC and represents a potential therapeutic target for future RNA-based interventions, including antisense oligonucleotides, locked nucleic acids, and microRNA mimics or inhibitors.

Epigenetic dysregulation reaches its most extreme and therapeutically refractory form in the epigenetic-senescent phenotype. This phenotype is characterized by persistent histone modifications (particularly H3K9 and H3K27 trimethylation at anti-calcific gene loci), widespread DNA hypermethylation (especially at MGP, GGCX, and autophagy gene promoters), and durable microRNA derangements that perpetuate vascular injury despite aggressive metabolic correction [14,27,28,29,30].

In this phenotypic state, conventional therapies targeting upstream metabolic triggers prove ineffective because the vascular reprogramming has become cell-autonomous and self-sustaining, locked in place by stable epigenetic modifications. Emerging therapeutic strategies for this phenotype include histone deacetylase (HDAC) inhibitors to reverse repressive chromatin marks, DNA methyltransferase inhibitors (DNMTis) to demethylate silenced protective genes, and targeted microRNA modulation to restore contractile gene programs. In contrast, in the inflammatory-oxidative phenotype, IS and PCS induce acute, potentially reversible epigenetic changes—transient histone acetylation, acute miR-30c suppression, and chromatin remodeling—that may respond to toxin removal via enhanced dialysis clearance coupled with antioxidant therapy and epigenetic stabilization strategies [12,37].

#### 5.2.4. Pharmacologically Induced or Functional Inhibitors

Beyond restoration of endogenous protective mechanisms, several pharmacologic agents aim to either mimic or potentiate physiological anti-calcific pathways, representing a paradigm of therapeutic biomimicry. SNF472, a synthetic myo-inositol hexaphosphate derivative (soluble phytate analog), functions as a direct crystal growth inhibitor by binding to nascent hydroxyapatite crystal surfaces and preventing their expansion, effectively freezing mineral deposits in a non-pathogenic, growth-arrested state. Clinical trials, most notably the CaLIPSO study, have demonstrated that SNF472 administration (300 mg intravenously three times weekly during hemodialysis) significantly reduces progression of both coronary artery and aortic valve calcification in dialysis patients compared to placebo [25]. SNF472 represents a mechanistically “universal” anti-calcific agent theoretically applicable across all phenotypes, as it acts at the terminal common pathway of hydroxyapatite crystallization. However, clinical efficacy appears greatest in the mineral-metabolic phenotype, where active crystal growth predominates due to persistent hyperphosphatemia and severe PPi depletion, and in the integrated toxic continuum, where conventional single-pathway therapies have failed and direct crystal growth inhibition offers a mechanism-independent intervention [25,75,76,77].

Vitamin K_2_ (menaquinone-7) reactivates matrix Gla protein through restoration of γ-carboxylation, converting the inactive dp-ucMGP form into functional, anti-calcific MGP. Clinical supplementation trials demonstrate reduction in circulating dp-ucMGP levels and modest improvements in vascular compliance, although results have been heterogeneous across studies [64]. Vitamin K_2_ is optimally suited for the epigenetic-senescent phenotype in its early stages (dialysis vintage < 3 years, dp-ucMGP elevation but without fully established epigenetic silencing) and for the endocrine cross-talk phenotype, where vitamin K deficiency results from gut dysbiosis, malabsorption, or drug interactions rather than epigenetic MGP pathway dysfunction. In advanced epigenetic-senescent states where GGCX and MGP genes are stably hypermethylated, vitamin K_2_ supplementation alone proves insufficient, and combination strategies coupling vitamin K_2_ with HDAC inhibitors or other epigenetic modulators to derepress the silenced carboxylation machinery represent a rational next-generation approach [64,65,66].

Magnesium-based phosphate binders (magnesium carbonate, magnesium hydroxide) and iron-based compounds (ferric citrate, sucroferric oxyhydroxide) provide dual functionality: phosphate binding to reduce mineral burden coupled with anti-calcific effects mediated through the properties of the cation itself. Magnesium stabilizes amorphous mineral phases, competes with calcium at nucleation sites, and exerts beneficial cellular effects on Wnt/β-catenin and eNOS pathways. Iron-based binders additionally modulate oxidative stress, stimulate autophagy in VSMCs, suppress osteochondrogenic microRNA networks (particularly miR-30c restoration), and reduce FGF23 production by correcting iron deficiency [55,59,62]. These dual-action agents show broad applicability but are particularly indicated in the mineral-metabolic phenotype, where both phosphate control and restoration of depleted inhibitory pathways (PPi, magnesium, autophagy) are critical to achieving therapeutic benefit.

### 5.3. Integration and Pathophysiological Balance

Ultimately, VC in CKD results from the catastrophic collapse of equilibrium between promotive forces and inhibitory defenses. This dynamic imbalance occurs when hyperphosphatemia, hormonal dysregulation (FGF23 excess, Klotho deficiency, PTH elevation), and chronic inflammation initiate and sustain osteogenic reprogramming of vascular cells, while simultaneous loss of Fetuin-A, MGP, PPi, and Klotho removes the intrinsic molecular brakes that normally prevent ectopic mineral deposition. These mechanisms operate not as isolated pathways but as interdependent loops wherein dysfunction in one system amplifies pathology in others. For example, systemic inflammation increases TNAP activity and depletes Fetuin-A; phosphate retention suppresses Klotho expression and amplifies FGF23 production; mitochondrial dysfunction generates ROS that drives both apoptosis and inflammatory signaling; and impaired autophagy permits accumulation of calcifying vesicles and damaged organelles that become intracellular nucleation sites. This pathogenic architecture is captured across the five mechanistic levels illustrated in Figure 2. Uremic toxins (Level 1) engage cellular receptors and stress sensors (Level 2), triggering intracellular signaling cascades (Level 3) that drive VSMC phenotypic transformation (Level 4) and ultimately tip the balance toward progressive vascular mineralization (Level 5) when inhibitory defenses are overwhelmed by pro-calcific drivers.

The vascular outcome—progressive calcification versus maintenance of vascular resilience—depends on the relative dominance and interaction of these competing forces. From the phenotypic perspective developed throughout this review, the specific pattern of promoter activation and inhibitor failure defines distinct vascular phenotypes with characteristic clinical presentations, biomarker profiles, imaging patterns, and therapeutic susceptibilities. The inflammatory- oxidative phenotype exhibits predominant toxin-driven inflammation with massive extracellular vesicle release and oxidative endothelial injury, showing preferential responsiveness to dialysis optimization (hemodiafiltration, expanded hemodialysis), toxin adsorbents, and anti-inflammatory strategies targeting NLRP3 or AhR pathways. The mineral-metabolic phenotype manifests maximal phosphate toxicity, FGF23 excess exceeding 200–500 RU/mL, profound Klotho deficiency, and severe PPi depletion, requiring intensive multi-level mineral axis control through phosphate binders, calcimimetics, direct crystal growth inhibitors (SNF472), and emerging TNAP inhibitors or ENPP1 enzyme replacement. The epigenetic-senescent phenotype demonstrates irreversible cellular reprogramming with stable chromatin modifications, persistent MGP pathway dysfunction despite vitamin K_2_ supplementation, and refractory calcification progression despite optimal metabolic control, necessitating cellular rejuvenation approaches including senolytic therapies, autophagy restoration, and potentially epigenetic reprogramming via HDAC inhibitors or DNA demethylating agents. The endocrine cross-talk phenotype manifests multisystem hormonal dysregulation extending beyond traditional CKD-MBD to encompass the kidney–bone–gut–vascular axis, with vitamin D deficiency, PTH resistance, FGF23-iron-EPO axis dysfunction, and intestinal dysbiosis, demanding integrated therapy addressing hormonal balance, microbiome optimization, and iron metabolism correction.

Finally, the integrated toxic continuum represents the convergence of all pathogenic pathways into a self-sustaining, self-amplifying toxic state where monotherapy targeting any single mechanism proves futile. Only multi-dimensional combination interventions addressing inflammation, mineral metabolism, cellular senescence, and endocrine dysfunction simultaneously—coupled with prioritization for kidney transplantation when feasible—offer realistic prospects for disease modification.

From a translational viewpoint, understanding this phenotypic framework provides the fundamental rationale for precision medicine approaches in VC therapeutics. Therapies must not only suppress promoters but also actively reinforce depleted physiological inhibitors, with both strategies matched to the patient’s dominant phenotypic drivers rather than applied uniformly across heterogeneous populations. This integrated view, linking molecular pathogenesis with phenotypic classification and precision therapeutic selection, defines the modern understanding of VC in CKD and establishes the conceptual foundation for the phenotype-guided intervention strategies discussed in the subsequent section. The transition from empiric, population-based biochemical correction to biomarker-driven, mechanism-matched, phenotype-informed precision therapy represents the translational frontier of uremic vascular medicine, transforming VC from an inevitable complication into a targetable manifestation of specific, identifiable toxic pathways amenable to rational, personalized intervention.

## 6. Phenotype-Guided Therapeutic Strategies for Uremic Toxin-Driven Vascular Calcification

### 6.1. From Empiric Biochemical Correction to Precision Vascular Medicine

**Figure 3:**
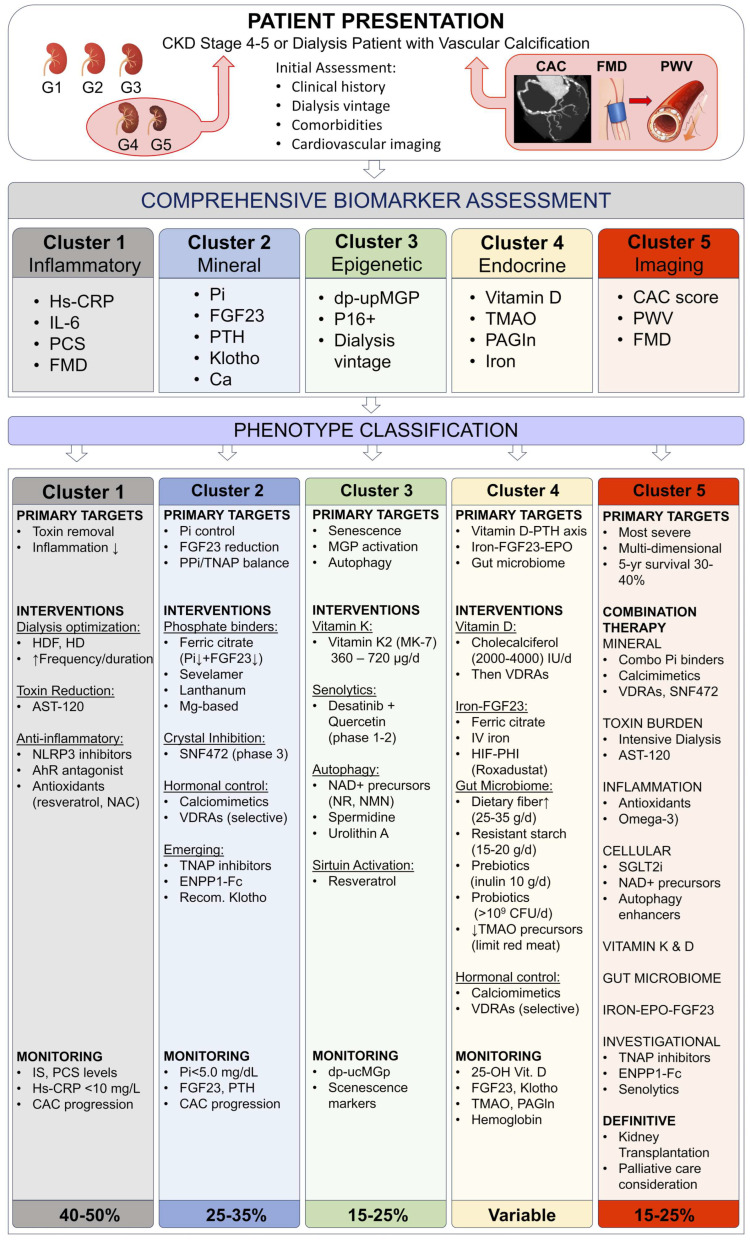
THERAPEUTIC ALGORITHM Precision-Based Therapeutic Selection for Uremic Toxin-Driven Vascular Calcification. ↓ indicates a decrease; ↑ indicates an increase.

The management of VC in CKD has evolved from straightforward biochemical correction of serum calcium, phosphate, and parathyroid hormone toward recognition of calcification as a dynamic, cell-mediated process driven by uremic toxins acting as active biological agents. These toxins reprogram vascular cells, disrupt protective mechanisms, and amplify inflammatory cascades. The specific constellation of toxin-driven perturbations defines distinct vascular phenotypes that respond differentially to therapeutic interventions [39,40,50] (Figure 3).

Effective implementation requires systematic biomarker-based stratification. The comprehensive panel in Table 2 encompasses uremic toxins, inflammatory mediators, mineral metabolism markers, and calcification inhibitors, enabling precise phenotypic classification and therapeutic monitoring. This transforms decision-making from population-based protocols to individualized, mechanism-based strategies matching interventions to each patient’s biological drivers.

Available therapeutic interventions are summarized in Table 3, categorized by mechanism of action, target phenotype, and evidence level. The following sections outline how specific modalities map to optimal phenotypic contexts, establishing a translational roadmap for precision-based management.

### 6.2. Targeting the Inflammatory-Oxidative Phenotype: Toxin Removal and Anti-Inflammatory Modulation

Patients with the inflammatory-oxidative phenotype—characterized by elevated protein-bound uremic toxins (IS > 100 μM, PCS > 150 μM), high-sensitivity C-reactive protein exceeding 10 mg/L, and severe endothelial dysfunction—require therapeutic strategies prioritizing toxin removal and suppression of inflammatory cascades.

Intensified dialysis modalities represent the cornerstone intervention. Online hemodiafiltration and expanded hemodialysis, utilizing high-flux membranes with enhanced middle-molecule clearance, demonstrate superior removal of indoxyl sulfate and p-cresyl sulfate compared to conventional hemodialysis, reducing circulating toxin levels by 20–30% [91]. Post hoc analyses from large dialysis cohorts suggest patients in the highest IS and PCS tertiles derive the greatest mortality benefit from these enhanced clearance strategies, supporting phenotype-specific therapeutic responsiveness [12,22,23,24]. Biomarker-guided identification of the inflammatory-oxidative phenotype should prompt consideration of dialysis modality optimization, particularly where hemodiafiltration or expanded hemodialysis is available.

Oral sorbents such as AST-120 bind indole precursors in the intestinal lumen, reducing IS generation. Although large Phase 3 trials (EPPIC-1, EPPIC-2) failed to demonstrate benefit in unselected CKD stage 3–5 populations, subgroup analyses revealed efficacy in patients with elevated baseline IS levels—precisely those manifesting the inflammatory-oxidative phenotype. This discordance between intention-to-treat results and biomarker-selected subgroup outcomes underscores a fundamental challenge: interventions with strong mechanistic rationale may fail in heterogeneous populations yet succeed in phenotypically enriched cohorts. AST-120 should be reconsidered for targeted application in high-IS phenotypes pending prospective validation in phenotype-selected trials.

Beyond mechanical toxin removal, targeting inflammatory and oxidative stress pathways activated by IS and PCS represents a rational complementary strategy. Natural antioxidants including resveratrol, N-acetylcysteine, and curcumin reduce reactive oxygen species and inhibit NF-κB signaling, attenuating osteogenic transformation of VSMCs in experimental models [73]. While large cardiovascular outcome trials in CKD are lacking, the mechanistic rationale and favorable safety profiles support their consideration as adjunctive therapies.

More transformative are emerging therapies targeting the NLRP3 inflammasome and aryl hydrocarbon receptor—the molecular mediators through which IS and PCS exert pro-inflammatory and pro-calcific effects. NLRP3 inflammasome inhibitors, currently in Phase 1–2 trials for gout and heart failure, selectively block IL-1β maturation, interrupting sterile inflammation driving vascular injury in uremia. Aryl hydrocarbon receptor antagonists directly inhibit transcriptional programs induced by IS and PCS, preventing NF-κB activation and oxidative stress at the receptor level. Preclinical data demonstrate both strategies attenuate uremia-induced VC and endothelial dysfunction. Their translation to phenotype-enriched clinical trials enrolling patients with elevated IS/PCS and inflammatory markers represents a high-priority precision therapy opportunity validating the phenotypic framework in prospective interventional studies.

### 6.3. Interventions for the Mineral-Metabolic Phenotype: Multi-Level Targeting of the Phosphate-Pyrophosphate Axis

The mineral-metabolic phenotype—persistent hyperphosphatemia, markedly elevated FGF23 (>200–500 RU/mL), secondary hyperparathyroidism, and profound Klotho deficiency—accounts for 40–50% of VC cases in dialysis populations and demands multi-level intervention across the phosphate-pyrophosphate homeostatic axis [3,25,32,85].

Phosphate retention drives osteogenic transdifferentiation of VSMCs via PiT-1/PiT-2 phosphate transporters and Runx2 transcriptional activation. Non-calcium-based phosphate binders (sevelamer, lanthanum carbonate, magnesium compounds) remain foundational, reducing intestinal phosphate absorption without adding exogenous calcium. Iron-based binders such as ferric citrate provide dual functionality: phosphate binding coupled with iron supplementation to address the iron-FGF23 axis. Iron deficiency stabilizes hypoxia-inducible factor-1α, promoting FGF23 transcription and cleavage. Conversely, iron repletion via ferric citrate reduces circulating FGF23 by 20–30% while exerting direct anti-calcific effects on vascular smooth muscle cells through modulation of the miR-30c network and autophagy enhancement [59,62,92]. This triple benefit—phosphate binding, FGF23 reduction, and direct vascular cytoprotection—positions ferric citrate as a precision therapeutic for mineral-metabolic phenotype patients with concurrent iron deficiency and elevated FGF23.

Management of secondary hyperparathyroidism through calcimimetics and selective vitamin D receptor activators addresses the hormonal component. Calcimimetics (cinacalcet, etelcalcetide) reduce PTH secretion via allosteric modulation of the calcium-sensing receptor, indirectly mitigating calcification progression by lowering serum calcium and phosphate. Selective vitamin D receptor activators (paricalcitol, doxercalciferol) suppress PTH while exerting pleiotropic anti-inflammatory, anti-proliferative, and endothelial protective effects through suppression of the renin–angiotensin–aldosterone system and NF-κB signaling [35,93,94]. The combination provides synergistic PTH control with more favorable calcium-phosphate profiles compared to monotherapy, representing the recommended strategy for severe secondary hyperparathyroidism.

At the leading edge are interventions directly targeting the pyrophosphate-alkaline phosphatase balance. SNF472, a myo-inositol hexaphosphate derivative, binds nascent hydroxyapatite crystal surfaces and prevents crystal growth, blocking the terminal calcification step. The CaLIPSO trial demonstrated that SNF472 (300 mg intravenously three times weekly during hemodialysis) significantly slowed progression of coronary artery and aortic valve calcifications in dialysis patients [25,75,76]. While SNF472 acts as a universal anti-calcific agent suitable across phenotypes, maximal benefit is expected in the mineral-metabolic phenotype where active crystal growth predominates due to persistent hyperphosphatemia and pyrophosphate depletion.

More mechanistically targeted are therapies addressing pyrophosphate deficiency: recombinant ENPP1-Fc enzyme replacement (INZ-701), which hydrolyzes extracellular ATP to generate pyrophosphate, and tissue-nonspecific alkaline phosphatase inhibitors, which prevent pyrophosphate degradation. Preclinical data demonstrate ENPP1-Fc reduces VC by 60% in CKD rat models while preserving bone architecture, and experimental TNAP inhibition with SBI-425 attenuates atherosclerotic plaque calcification without inducing osteomalacia [68,69,70,71]. Phase 2 trials of ENPP1-Fc in phenotype-enriched CKD populations—specifically patients with FGF23 >500 RU/mL and Klotho <300 pg/mL—are anticipated within two years and represent a transformative precision therapy opportunity for the mineral-metabolic phenotype.

### 6.4. Addressing the Epigenetic-Senescent Phenotype: Cellular Reprogramming Strategies

The epigenetic-senescent phenotype emerges in patients with prolonged dialysis vintage (typically exceeding five years) and is characterized by paradoxical calcification progression despite optimal phosphate control and adequate dialysis clearance, reflecting irreversible vascular reprogramming that conventional metabolic therapies cannot reverse [14,16,27,28,29,30]. The biomarker signature includes persistently elevated dephosphorylated-uncarboxylated matrix Gla protein (>1500–2000 pmol/L), markers of cellular senescence (circulating p16^INK4a^-positive cells, increased senescence-associated β-galactosidase activity), and evidence of systemic oxidative damage. This indicates underlying drivers are no longer biochemical but cellular, reflecting fundamental transformation of vascular cell identity from contractile to senescent-osteogenic.

For this phenotype, vitamin K_2_ supplementation—which activates matrix Gla protein through γ-carboxylation—shows limited efficacy despite mechanistic appeal. Clinical trials enrolling long-term dialysis patients demonstrate that vitamin K_2_ reduces dephosphorylated-uncarboxylated MGP levels yet fails to halt calcification progression, likely because the MGP carboxylation pathway itself is epigenetically silenced through persistent DNA methylation at the gamma-glutamyl carboxylase gene promoter and histone deacetylation at MGP regulatory regions [14,27,64,65,66]. This functional vitamin K resistance highlights a critical lesson: interventions effective in early-stage disease lose efficacy once the epigenetic-senescent phenotype is established, underscoring the importance of early phenotypic identification and preventive intervention before cellular reprogramming becomes irreversible.

The therapeutic frontier for established epigenetic-senescent phenotype lies in cellular reprogramming strategies. Senolytic therapies—drugs that selectively eliminate senescent cells while sparing healthy cells—represent the most advanced approach. Dasatinib combined with quercetin induces apoptosis in p16^INK4a^-high senescent cells via inhibition of BCL-2 family anti-apoptotic proteins. Preclinical data in aged mice and atherosclerosis models demonstrate that dasatinib-quercetin reduces vascular senescent cell burden by 50–70%, decreases arterial stiffness, and attenuates aortic calcification. Early human trials in idiopathic pulmonary fibrosis and diabetic kidney disease show safety and preliminary efficacy, with reduction in circulating senescence-associated secretory phenotype markers [29]. While no trials have specifically enrolled the epigenetic-senescent CKD VC phenotype, the mechanistic rationale is compelling, and phenotype-enriched trials (dialysis vintage > 5 years, dp-ucMGP > 2000 pmol/L, high p16^ + ^ cell burden) represent a high-priority translational opportunity.

Complementary to senolytic elimination is restoration of cellular homeostasis through autophagy enhancement. Spermidine, urolithin A, and nicotinamide riboside (NAD^ + ^ precursor) induce autophagy and mitophagy, facilitating clearance of damaged organelles and calcifying vesicles while activating sirtuins (SIRT1/3) that regulate mitochondrial integrity and cellular senescence [29,73,74]. These agents, with favorable safety profiles and human aging trial data supporting efficacy in restoring cellular function, offer adjunctive or synergistic benefit when combined with senolytics.

However, current cellular reprogramming approaches remain investigational, with limited clinical validation in CKD populations. Patients should be counseled regarding the refractory nature of their condition, the experimental status of these therapies, and the importance of prioritization for kidney transplantation—which, by restoring overall renal function and normalizing the uremic milieu, remains the most definitive intervention capable of halting disease progression and, in some cases, achieving modest regression of vascular calcification.

### 6.5. Endocrine Cross-Talk and Gut–Vascular Axis: Integrated Hormonal and Microbiome Interventions

The endocrine cross-talk phenotype manifests as multisystem hormonal dysregulation extending beyond traditional CKD-mineral and bone disorder to encompass the kidney-bone-vascular axis, gut–vascular interface, and broader neuroendocrine perturbations [31,32,33,35,36]. Patients typically present with profound vitamin D deficiency (25-hydroxyvitamin D <20 ng/mL), Klotho deficiency, FGF23 elevation with evidence of FGF23 resistance, and secondary hyperparathyroidism resistant to conventional therapy. Concurrently, these patients exhibit elevated gut-derived uremic toxins—particularly trimethylamine N-oxide, phenylacetylglutamine, and indole derivatives—reflecting intestinal dysbiosis with depletion of beneficial short-chain fatty acid-producing bacteria and expansion of proteolytic species.

Therapeutic management demands integrated intervention across multiple endocrine axes. Vitamin D repletion through nutritional supplementation (cholecalciferol 2000–4000 IU daily or calcifediol 30 μg daily) should target 25-hydroxyvitamin D levels exceeding 30 ng/mL, followed by selective vitamin D receptor activators (paricalcitol, doxercalciferol) to suppress PTH and exert pleiotropic anti-inflammatory and cardiovascular protective effects while respecting the narrow therapeutic window avoiding paradoxical hypercalcemia-induced calcification [34,95,96,97].

Optimization of the iron-erythropoietin-FGF23 axis through iron supplementation (preferably oral ferric citrate for dual phosphate-binding and iron-provision benefits, or intravenous iron if oral intolerance) reduces FGF23 production by correcting iron deficiency and HIF-1α stabilization, breaking the vicious cycle wherein FGF23 excess suppresses erythropoietin production and causes EPO resistance [35,36,37,38,90,94,97]. Hypoxia-inducible factor-prolyl hydroxylase inhibitors (roxadustat, daprodustat), which stimulate endogenous erythropoietin production and enhance iron absorption while potentially reducing FGF23 levels, represent an emerging alternative to traditional erythropoiesis-stimulating agents.

Modulation of the gut microbiome to reduce uremic toxin generation and restore beneficial metabolite production is equally critical. Dietary interventions emphasizing increased fiber intake (25–35 g daily from vegetables, fruits, whole grains, legumes) should be supplemented with prebiotics (resistant starch 15–20 g daily, inulin 10 g daily) and probiotics (multi-strain formulations containing *Bifidobacterium* and *Lactobacillus* species at ≥10 billion colony-forming units daily), shifting intestinal microbiota toward saccharolytic rather than proteolytic metabolism. Small CKD trials demonstrate that resistant starch supplementation reduces serum indoxyl sulfate by 30%, lowers inflammatory markers, and improves gut microbiome diversity [33,90]. Dietary reduction of TMAO precursors (limiting red meat, eggs, high-fat dairy) further decreases production of this proatherogenic and pro-calcific gut-derived metabolite.

While dietary fiber, prebiotics, and probiotics represent evidence-based approaches with favorable safety profiles, emerging therapies targeting microbial trimethylamine production (3,3-dimethyl-1-butanol inhibitors) and fecal microbiota transplantation remain investigational with limited clinical validation in CKD populations and should be considered only within research protocols.

### 6.6. Multi-Target Strategies for the Integrated Toxic Continuum

The integrated toxic continuum represents the convergence point where inflammatory, metabolic, epigenetic, and endocrine pathways merge into a self-sustaining state of systemic toxicity, characterized by simultaneous derangements across all biomarker domains: severe hyperphosphatemia, markedly elevated FGF23 (>500–1000 RU/mL), profound Klotho deficiency, PTH exceeding 800–1000 pg/mL, high indoxyl sulfate and p-cresyl sulfate, systemic inflammation (hs-CRP > 10 mg/L), and extensive VC burden (coronary artery calcium Agatston score > 400, often >1000) [16,37,38,39,40,41]. Patients exhibit diffuse cardiovascular calcification affecting coronary arteries, aorta, peripheral vessels, and cardiac valves, accompanied by severe arterial stiffness (pulse wave velocity > 12–15 m/s) and left ventricular hypertrophy. Critically, these patients demonstrate minimal or paradoxical responses to single-pathway interventions because the pathogenic network has acquired self-reinforcing properties.

The therapeutic approach must be multi-dimensional, prioritizing interventions that target mineral metabolism through combination phosphate binders (ferric citrate, sevelamer, magnesium compounds), high-dose calcimimetics, selective vitamin D receptor activators, and SNF472 if available, while intensifying uremic toxin removal via expanded dialysis modalities (hemodiafiltration with increased frequency or extended hours). Additional interventions should address inflammation and oxidative stress (antioxidants including resveratrol, N-acetylcysteine, curcumin, omega-3 fatty acids), cellular homeostasis (SGLT2 inhibitors, NAD^ + ^ precursors, spermidine, urolithin A to enhance autophagy and mitochondrial function), vitamin K and inhibitor repletion (vitamin K_2_, vitamin D optimization), gut microbiome modulation (prebiotics, probiotics, dietary fiber), and iron-erythropoietin-FGF23 axis optimization. This comprehensive regimen reflects the biological reality that the integrated continuum has transcended single-pathway dominance and requires restoration of homeostasis across multiple axes simultaneously.

For patients with refractory disease despite maximal conventional treatment, investigational add-on therapies—including TNAP inhibitors, ENPP1-Fc replacement, senolytic therapy, NLRP3 inflammasome inhibitors, and recombinant Klotho—remain experimental with limited clinical validation in CKD populations. Clinical trial enrollment should be pursued when feasible, as these represent cutting-edge precision approaches specifically suited for phenotypes resistant to standard therapies.

The integrated toxic continuum represents advanced uremic vascular pathology where conventional prevention has been insufficient and restoration of vascular integrity may require transformative approaches, foremost among them kidney transplantation. Patients with this phenotype should be expedited for transplant evaluation and listing, accepting even marginal donor organs (extended criteria donors, donation after cardiac death) given the poor prognosis on dialysis (5-year survival 30–40%). Successful transplantation, by fully restoring kidney function and normalizing the uremic milieu, remains the most definitive intervention capable of halting calcification progression and, in select cases, achieving modest regression. For patients ineligible for transplantation due to age, comorbidities, or contraindications, honest prognostic discussions acknowledging the refractory nature of their condition, integration of palliative care alongside aggressive medical therapy, and focus on quality of life and symptom management become paramount.

### 6.7. Emerging Therapies and Translational Horizons

The therapeutic pipeline for VC in CKD is rapidly expanding with mechanism-based agents mapping to specific phenotypic contexts. Crystal growth inhibitors such as SNF472, already demonstrating clinical efficacy and approaching regulatory approval, represent universal anti-calcific agents blocking terminal mineralization regardless of upstream drivers, with maximal benefit anticipated in mineral-metabolic and integrated continuum phenotypes where active crystallization predominates. Enzymatic therapies targeting the pyrophosphate-alkaline phosphatase axis—recombinant ENPP1-Fc enzyme replacement and tissue-nonspecific alkaline phosphatase inhibitors—address the root cause of pyrophosphate depletion and are optimally suited for the mineral-metabolic phenotype characterized by FGF23 excess, Klotho deficiency, and severe pyrophosphate depletion [68,69,70,71].

Anti-inflammatory therapies specifically targeting uremic pathways—NLRP3 inflammasome inhibitors and aryl hydrocarbon receptor antagonists—are designed for the inflammatory-oxidative phenotype, offering selective interruption of indoxyl sulfate and p-cresyl sulfate-mediated inflammatory cascades. Senolytic and anti-senescence therapies aim to eliminate or reprogram senescent vascular cells and are uniquely suited for epigenetic-senescent and integrated continuum phenotypes where cellular senescence drives refractory calcification despite metabolic correction [29]. Vitamin K_2_ and next-generation MGP activators target the endocrine cross-talk and early epigenetic-senescent phenotypes, though efficacy is limited once irreversible chromatin remodeling is established [64,65,66]. FGF23-Klotho axis modulators—FGF23 neutralizing antibodies, FGFR antagonists that selectively block off-target cardiotoxic effects while preserving phosphaturic actions, and recombinant soluble Klotho—are rational precision targets for mineral-metabolic and endocrine cross-talk phenotypes characterized by profound FGF23-Klotho imbalance [32,72].

The critical translational principle underlying this therapeutic landscape is phenotype-based patient selection. The failure of several promising therapies in Phase 3 trials—AST-120, vitamin K_2_, various phosphate binders when tested for cardiovascular outcomes—can be largely attributed to enrollment of phenotypically heterogeneous populations wherein only a subset possessed the biological characteristics rendering them responsive to the mechanism of action. Future clinical trial design must embrace phenotypic enrichment, enrolling patients whose biomarker profiles match the therapeutic mechanism: high indoxyl sulfate and p-cresyl sulfate with inflammatory markers for NLRP3 or aryl hydrocarbon receptor inhibitors; elevated FGF23, hyperphosphatemia, and Klotho deficiency for ENPP1-Fc or TNAP inhibitors; long dialysis vintage, elevated dp-ucMGP, and senescence markers for senolytics; gut dysbiosis with TMAO elevation for microbiome modulators. This precision trial paradigm, integrating biomarker-driven patient stratification with mechanism-based intervention and composite imaging-biomarker-clinical endpoints, transforms VC research from empiric population-based testing to hypothesis-driven precision investigation where therapeutic efficacy is maximized by matching drugs to their optimal biological contexts.

### 6.8. Relationship to Current Guidelines

The KDIGO 2017 Clinical Practice Guideline Update for the Diagnosis, Evaluation, Prevention, and Treatment of Chronic Kidney Disease-Mineral and Bone Disorder [98], reaffirmed in the 2023 KDIGO Controversies Conference on CKD-MBD [99] and the KDIGO 2024 Clinical Practice Guideline for the Evaluation and Management of CKD [100], remains the evidence-based foundation for managing mineral metabolism disturbances and vascular calcification. KDIGO recommendations—phosphate control through dietary restriction and non-calcium-based binders, parathyroid hormone management with calcimimetics and vitamin D receptor activators, and avoidance of calcium-based therapies in the setting of vascular calcification—represent the standard of care supported by randomized controlled trial evidence.

The phenotypic classification system presented here operates at a complementary level, providing a mechanistic lens to interpret biological heterogeneity underlying individual patient responses to guideline-concordant therapy. While KDIGO guidelines establish population-level treatment targets applicable to all patients with CKD-MBD, the phenotypic framework enables clinicians to understand why some patients achieve adequate biochemical control yet continue calcification progression (epigenetic-senescent phenotype), why others develop vascular injury despite normal phosphate levels (inflammatory-oxidative phenotype), and why therapeutic modalities demonstrate variable efficacy across patient subgroups.

This framework does not advocate deviation from evidence-based guidelines but proposes an additional layer of biological insight informing therapeutic prioritization within guideline-recommended options. For instance, a patient with mineral-metabolic phenotype exhibiting elevated fibroblast growth factor 23 and profound Klotho deficiency might benefit from preferential use of iron-based phosphate binders given dual effects on phosphate binding and FGF23 reduction. Similarly, a patient manifesting inflammatory-oxidative phenotype with markedly elevated protein-bound uremic toxins might derive greater benefit from intensified dialysis modalities compared to conventional schedules, even when meeting current adequacy targets.

As evidence matures through phenotype-enriched clinical trials, the framework may eventually inform refinements to future guideline iterations, potentially enabling precision medicine recommendations where specific interventions are preferentially indicated for biomarker-defined patient subgroups. Until such validation is achieved, the phenotypic approach should be viewed as a hypothesis-generating research tool and educational framework for understanding uremic vascular pathophysiology, rather than a departure from established clinical standards.

## 7. Future Perspectives: From Phenotypic Discovery to Precision Clinical Trials

Research on VC in CKD is entering a transformative era, marked by the shift from prevention-focused biochemical correction to active vascular intervention guided by phenotypic stratification. The evolving landscape of clinical trials targeting VC in CKD is summarized in Table 4. The traditional view of VC as an irreversible degenerative process is being replaced by the concept of a dynamic, biologically regulated phenomenon—one that can potentially be stabilized, modulated, and even reversed through targeted molecular and cellular therapies matched to individual pathogenic profiles. This paradigm shift mirrors the evolution seen in oncology and cardiology, where the focus has moved from population-based risk mitigation to precision medicine anchored in molecular and cellular diagnostics. In CKD, this means that the new therapeutic frontier is no longer confined to phosphate control and biochemical normalization but aims to reestablish a functional equilibrium between calcification promoters and inhibitors, thereby restoring the physiological resilience of the vascular wall through phenotype-informed therapeutic selection.

Current clinical trials registered on ClinicalTrials.gov and in academic pipelines depict an increasingly sophisticated landscape of innovation. Among these, the SNF472 development program (NCT02966028; NCT04195906) stands as a landmark milestone in crystal-targeted therapy. Derived from myo-inositol hexaphosphate, SNF472 selectively binds to the surface of hydroxyapatite crystals, preventing their growth and stabilizing amorphous, non-pathogenic Ca-Pi complexes. In hemodialysis patients, this agent demonstrated a significant reduction in the progression of coronary and valvular calcifications in the CaLIPSO trial [25,75,76], thus validating the concept that direct inhibition of the terminal mineralization pathway can achieve clinical benefit. Notably, post hoc analyses suggest greatest efficacy in patients with biochemical features consistent with the mineral-metabolic phenotype—elevated FGF23, hyperphosphatemia, and Klotho deficiency—highlighting how even “universal” crystal inhibitors may exhibit phenotype-dependent responsiveness.

In parallel, several ongoing studies are exploring nutritional and metabolic interventions aimed at restoring mineral balance and enhancing endogenous anti-calcific mechanisms. Trials investigating magnesium supplementation (NCT02542319), vitamin K_2_ (menaquinone-7) therapy (NCT04539418), and modified dialysate composition (NCT07163936) each address specific facets of the calcification network with distinct mechanistic rationales. Magnesium acts as a physicochemical stabilizer of amorphous mineral phases while simultaneously counteracting Wnt/β-catenin signaling and promoting endothelial nitric oxide synthesis [55]. Vitamin K_2_ reactivates matrix Gla protein through γ-carboxylation, converting it from an inactive to an active anti-calcific form [64,65,66]. Optimized dialysate calcium and phosphate concentrations modulate the vascular burden of calcifying substrates directly at the blood-vessel interface. Yet despite mechanistic plausibility, results from vitamin K trials have been heterogeneous, with benefit most evident in early-stage CKD and minimal effect in long-term dialysis patients. This discordance likely reflects phenotypic heterogeneity, with vitamin K_2_ effective in the endocrine cross-talk phenotype but ineffective once the epigenetic-senescent phenotype has been established [14,27,28,29,30].

Beyond nutritional and dialytic strategies, the research horizon is rapidly expanding toward molecular and enzymatic biotherapies. The recombinant enzyme ENPP1-Fc (INZ-701) represents a first-in-class approach designed to restore circulating pyrophosphate levels and thus reinforce one of the body’s most potent natural inhibitors of calcification [70,71]. Initially developed for rare genetic deficiencies (ENPP1 or ABCC6 mutations causing generalized arterial calcification of infancy and pseudoxanthoma elasticum), this therapeutic concept is now being considered for broader application in advanced CKD, where acquired pyrophosphate depletion mimics genetic ENPP1 deficiency. Preclinical data in rat CKD models demonstrate that ENPP1-Fc reduces VC by 60% while preserving normal bone architecture, addressing a critical concern in anti-calcific therapy [70]. Phase 2b trials in phenotype-enriched CKD populations—specifically, patients with the mineral-metabolic phenotype characterized by FGF23 > 500 RU/mL and Klotho < 300 pg/mL—are anticipated within the next two years and represent a high-priority translational milestone.

In parallel, TNAP inhibition—as demonstrated in preclinical studies with the small molecule SBI-425—has emerged as a highly promising strategy to rebalance the PPi/Pi ratio and protect vascular tissues without impairing bone mineralization [68,69]. Goettsch and colleagues established TNAP as a key nodal regulator of the phosphate-pyrophosphate equilibrium in the vasculature, and experimental TNAP inhibition reduced atherosclerotic plaque calcification while preserving skeletal integrity in ApoE-deficient mice [68]. These enzyme-targeted approaches epitomize the ongoing transition from empiric to mechanistic precision therapy, in which the vascular wall is no longer a passive bystander but an active therapeutic target.

Despite these advances, several barriers continue to limit the full translation of experimental discoveries into clinical impact. Most ongoing trials still rely on surrogate endpoints, such as coronary artery calcium scores or pulse wave velocity, which, although highly sensitive to changes in calcification burden, do not always predict hard cardiovascular outcomes such as myocardial infarction, stroke, or cardiovascular death. While progression of CAC correlates with mortality in observational studies, the causal link between slowing CAC progression and improving survival remains incompletely validated in interventional trials. A further challenge lies in the phenotypic heterogeneity of CKD-associated vascular calcification, encompassing inflammatory-oxidative, mineral-metabolic, epigenetic-senescent, endocrine cross-talk, and integrated toxic continuum patterns, each characterized by distinct molecular signatures, clinical trajectories, and therapeutic susceptibilities. This diversity underscores the critical need for stratified and phenotype-driven trial design, where patient selection criteria and therapeutic endpoints reflect the underlying biological mechanisms rather than uniform biochemical targets.

The failure of several promising therapies in Phase 3 trials—most notably AST-120 (EPPIC trials), vitamin K_2_ supplementation (heterogeneous results across trials), and various phosphate binders (when tested for cardiovascular outcomes rather than surrogate biochemical endpoints)—can be largely attributed to enrollment of phenotypically heterogeneous populations. For instance, AST-120 failed to demonstrate benefit in unselected CKD stages 3–5 populations, yet post hoc analyses revealed efficacy in patients with elevated baseline indoxyl sulfate levels—precisely those presenting with the inflammatory-oxidative phenotype [12,22]. Similarly, vitamin K_2_ trials enrolling long-term dialysis patients (>5 years vintage) showed minimal benefit despite normalization of dephosphorylated-uncarboxylated MGP, likely because these patients had transitioned to the epigenetic-senescent phenotype where MGP pathway dysfunction is irreversibly locked by chromatin remodeling [14,27,28,29,30]. These examples underscore a fundamental principle: therapeutic efficacy is not solely a property of the drug itself but reflects the match between the drug’s mechanism and the patient’s dominant pathogenic drivers.

An equally critical methodological challenge is the bone-vascular balance, an ever-present concern in CKD-MBD therapeutics. Any attempt to suppress vascular mineralization must avoid compromising skeletal integrity, a delicate equilibrium given that bone and vascular systems are governed by shared osteogenic pathways and molecular mediators (Runx2, BMP-2, ALP, MGP). Agents such as bisphosphonates or TNAP inhibitors, while effective in preventing ectopic calcification, carry theoretical risks of adynamic bone disease or osteomalacia if systemic inhibition is excessive. Future therapies must therefore be calibrated to achieve vascular selectivity—either through targeted delivery systems (e.g., calcification-targeting nanoparticles), dose optimization that preserves bone TNAP activity while inhibiting vascular TNAP, or exploitation of differential regulatory environments between bone and vascular tissues. The successful development of ENPP1-Fc, which restores PPi without inducing osteomalacia, demonstrates that such a balance is achievable [70,71].

Furthermore, progress in this field is hampered by the lack of standardized biomarkers to quantify calcification risk, classify phenotypic patterns, and monitor therapeutic efficacy. Circulating markers such as plasma pyrophosphate, dephosphorylated-uncarboxylated matrix Gla protein (dp-ucMGP), soluble Klotho, intact and C-terminal FGF23, indoxyl sulfate, p-cresyl sulfate, and senescence-associated secretory phenotype (SASP) factors remain underutilized in clinical trial design and are rarely incorporated as inclusion criteria or stratification variables. Establishing standardized assays with validated cut-offs for each biomarker, alongside consensus definitions of each phenotypic cluster, would greatly enhance comparability across studies, enable phenotype-enriched enrollment, and facilitate more precise monitoring of vascular responses to therapy. The integration of imaging biomarkers—coronary artery calcium scoring by CT, intravascular ultrasound for early microcalcification detection, ^18^F-sodium fluoride PET for active mineralization, and pulse wave velocity for functional vascular stiffness—with circulating biomarkers would provide a comprehensive phenotypic and prognostic assessment platform.

Looking ahead, the most promising horizon lies in combined and modular therapeutic strategies capable of acting on multiple pathogenic mechanisms simultaneously, tailored to the patient’s phenotypic profile. For the inflammatory-oxidative phenotype, the association of intensified dialysis modalities (hemodiafiltration, expanded hemodialysis) with antioxidant and anti-inflammatory agents (resveratrol, N-acetylcysteine, investigational NLRP3 or AhR inhibitors) targets the primary drivers of toxin-mediated oxidative vascular injury. For the mineral-metabolic phenotype, combining phosphate binders (preferably iron-based such as ferric citrate to address concurrent FGF23 elevation) with calcimimetics, SNF472 crystal growth inhibition, and emerging ENPP1 or TNAP-targeted therapies offers multi-level intervention across the phosphate-pyrophosphate axis. For the epigenetic-senescent phenotype, the integration of senolytic agents (dasatinib + quercetin), autophagy enhancers (spermidine, urolithin A, NAD^+^ precursors), and potentially epigenetic modulators (HDAC inhibitors) addresses the irreversible cellular reprogramming that conventional metabolic therapies cannot reverse. For the endocrine cross-talk phenotype, optimizing vitamin D status, modulating the gut microbiome with prebiotics and probiotics, and addressing the iron-EPO-FGF23 axis targets the complex hormonal dysregulation spanning the kidney–bone–gut–vascular network. Finally, for the integrated toxic continuum—representing the convergence of all pathogenic pathways in advanced disease—multi-dimensional combination therapy addressing inflammation, mineral metabolism, cellular senescence, and endocrine dysfunction simultaneously, alongside prioritization for kidney transplantation when feasible, offers the only realistic approach to disease modification.

This integrative, phenotype-matched therapeutic paradigm aligns with the broader principles of systems medicine, aiming to reprogram the calcification network rather than simply attenuate its downstream consequences. The next generation of clinical research will likely embrace adaptive and biomarker-guided trial designs, integrating high-throughput imaging (serial CAC and PWV assessments), multi-omics profiling (genomics, transcriptomics, metabolomics to refine phenotypic classification), and machine learning algorithms to dynamically assess vascular response and predict treatment responsiveness. Such an approach could identify patient subgroups most likely to benefit from specific interventions, transforming VC management from empiric population-based therapy to precision nephrology anchored in individual biological signatures. Importantly, longer-term follow-up studies with composite hard cardiovascular endpoints—myocardial infarction, stroke, heart failure hospitalization, cardiovascular death—are needed to validate whether modulation of calcification surrogates (CAC scores, PWV) translates into true reductions in morbidity and mortality, the ultimate validation of therapeutic value.

Ultimately, the field is moving from a paradigm of “biochemical value-correction medicine”—focused solely on restoring serum calcium, phosphate, and PTH levels to target ranges—to a new vision of “phenotypic vascular resilience medicine,” grounded in cellular repair, molecular homeostasis, and personalized therapeutic selection. In this emerging framework, the vessel wall is reconceptualized as a dynamic therapeutic organ, responsive to pharmacologic modulation and capable of regaining homeostatic function once pro-calcific stressors are neutralized through phenotype-matched interventions. The convergence of molecular mechanistic insight, advanced phenotypic diagnostics, translational innovation in drug development, and sophisticated trial methodology now makes this goal more attainable than ever. The phenotypic classification framework proposed herein—encompassing inflammatory-oxidative, mineral-metabolic, epigenetic-senescent, endocrine cross-talk, and integrated toxic continuum clusters—provides both a conceptual foundation for understanding VC heterogeneity and a practical roadmap for clinical trial design, biomarker-guided patient selection, and precision therapeutic implementation. As this vision is translated into clinical practice and research protocols, VC in CKD may transition from an inevitable complication of kidney failure to a targetable, potentially reversible manifestation of uremic toxicity, amenable to phenotype-informed precision intervention.

## 8. Validation Roadmap: From Hypothesis to Clinical Implementation

Translation of this phenotypic framework from conceptual hypothesis to validated clinical tool requires systematic validation across multiple investigational phases: unsupervised biomarker clustering, prospective outcome validation, and phenotype-stratified therapeutic trials.

Phase 1: Unsupervised Clustering and Phenotype Discovery

The first critical step involves applying unsupervised machine learning algorithms—hierarchical clustering, k-means clustering, and latent class analysis—to comprehensive multi-biomarker datasets from well-characterized chronic kidney disease cohorts. These analyses should integrate the proposed biomarker panel (uremic toxins, mineral metabolism markers, inflammatory mediators, calcification inhibitors, vascular function parameters) without a priori phenotypic assignments. If the five proposed phenotypes represent true biological entities, unbiased clustering algorithms should independently identify similar patient groupings. Discordance between hypothesis-driven and data-driven classifications would necessitate framework revision, while concordance would provide empirical support for phenotypic validity. Large existing cohorts with banked biospecimens and imaging data—including the Chronic Renal Insufficiency Cohort, the German Chronic Kidney Disease study, and national dialysis registry biobanks—represent ideal populations for validation studies.

Phase 2: Prospective Outcome Studies

Following cluster validation, prospective longitudinal studies must demonstrate that phenotypic classification provides clinically meaningful prognostic information beyond established risk factors. Phenotype assignment should independently predict differential rates of VC progression (quantified by serial coronary artery calcium scoring or pulse wave velocity), cardiovascular events, and all-cause mortality. Demonstration that phenotype-specific biomarker signatures confer incremental prognostic value would justify incorporation into risk stratification algorithms and clinical decision support tools.

Phase 3: Phenotype-Stratified Therapeutic Trials

Ultimate validation requires demonstrating differential therapeutic responses across phenotypes through prospective randomized controlled trials with phenotype-based enrollment criteria. Examples include trials of NLRP3 inflammasome inhibitors or aryl hydrocarbon receptor antagonists enrolling patients with elevated indoxyl sulfate and inflammatory markers (inflammatory-oxidative phenotype); ENPP1-Fc enzyme replacement or tissue-nonspecific alkaline phosphatase inhibitors in patients with elevated fibroblast growth factor 23 and depleted Klotho (mineral-metabolic phenotype); and senolytic agents in long-term dialysis patients with elevated dephosphorylated-uncarboxylated matrix Gla protein and senescence markers (epigenetic-senescent phenotype). Demonstration that phenotype-selected populations exhibit superior therapeutic responses compared to unselected cohorts would validate the precision medicine paradigm and transform this framework from theoretical construct to actionable clinical tool.

## 9. Conclusions: Vascular Calcification as a Targetable Toxic Phenotype

VC in CKD, once regarded as an inexorable degenerative process, is now recognized as an actively regulated, cell-mediated biological phenomenon governed by complex molecular networks responsive to mechanism-based intervention. Advances ranging from direct crystal-growth inhibitors to recombinant enzymes restoring pyrophosphate homeostasis, metabolic reprogramming agents, and emerging senolytic therapies collectively demonstrate that the calcifying vasculature represents a dynamic therapeutic target rather than a static endpoint.

Central to this paradigm shift is recognition that VC is not a monolithic entity but a heterogeneous spectrum of toxin-driven phenotypes, each reflecting distinct biological trajectories with characteristic molecular signatures and therapeutic vulnerabilities. The five mechanistic-clinical clusters described, inflammatory-oxidative, mineral-metabolic, epigenetic-senescent, endocrine cross-talk, and integrated toxic continuum, provide a translational framework bridging molecular pathophysiology with clinical decision-making. By linking specific biomarker profiles to distinct pathogenic mechanisms and corresponding therapeutic targets, clinicians can move beyond empiric population-based protocols toward individualized interventions tailored to each patient’s dominant drivers of vascular injury.

This phenotypic framework requires prospective validation through comprehensive biomarker assessment in large chronic kidney disease cohorts, phenotype-enriched randomized controlled trials demonstrating differential therapeutic responses, and longitudinal studies confirming prognostic value beyond established risk factors. Implementation challenges including biomarker availability, assay standardization, and resource constraints necessitate development of simplified phenotyping algorithms. Phenotype boundaries are not absolute, patients may exhibit overlapping features requiring hierarchical therapeutic prioritization. Despite these limitations, the framework establishes a translational roadmap for precision vascular medicine in chronic kidney disease, transforming VC from an inevitable complication into a targetable manifestation of uremic toxicity amenable to early detection, phenotypic classification, and precision-guided intervention.

## Figures and Tables

**Table 1:** Mechanistic-Clinical Clusters of Uremic Toxin-driven Vascular Calcification in Chronic Kidney Disease.

Phenotypic Cluster	Dominant Uremic Toxins	Key Molecular Pathways	Clinical Biomarkers	Typical Patient Profile	Primary Therapeutic Targets	Evidence Level
Inflammatory–Oxidative	IS, PCS, AGEs, TMAO, kynurenine	AhR → NF-κB → NLRP3; NADPH oxidase; ROS generation; EndMT; p38 MAPK/JNK activation	hs-CRP > 10 mg/L; IL-6, IL-1β ↑; FMD < 5%; Microcalcifications on IVUS; IS > 100 μM, PCS > 50 μM	Hemodialysis patients; Diabetes; Protein-energy wasting; Younger age with rapid VC progression; High inflammatory burden	AhR antagonists; Antioxidants (NAC, resveratrol); NLRP3 inhibitors; Enhanced dialytic removal of protein-bound toxins	★★
Mineral–Metabolic	Phosphate (Pi), FGF23, calcium excess	Pi → PiT-1/2 → ERK1/2/Smad → Runx2 activation; FGF23–Klotho axis disruption; ↓ PPi (ENPP1/ANK deficiency); ↑ TNAP activity; BMP2/4–Wnt/β-catenin signaling	Serum Pi > 5.5 mg/dL; FGF23 > 200 RU/mL; PTH > 600 pg/mL; Klotho ↓; CAC score progression; ↑ PWV	Advanced CKD (stage 4–5); Dialysis with poor Pi control; sHPT; Medial (Mönckeberg) calcification; Progressive arterial stiffness	Non-Ca phosphate binders (sevelamer, lanthanum); Iron-based binders (ferric citrate); Calcimimetics; TNAP inhibitors; SNF472 (crystal growth blocker)	★★★
Epigenetic–Senescent	IS, PCS (chronic exposure)	HDAC1/2 inhibition; ↑ Histone acetylation at Runx2/Msx2 promoters; ↓ miR-125b, miR-143/145, miR-204; ↑ miR-92a, miR-223; p53/p21 activation; SASP; Mitochondrial dysfunction	dp-ucMGP ↑; Senescence markers (p16, p21); mtDNA damage; Low autophagy flux (LC3-II/I ratio); Persistent VC despite biochemical correction	Long-term dialysis (>5 years); Older patients; VC refractory to standard treatment; “Molecular memory” of vascular injury; Progressive despite Pi control	HDAC modulators; Sirtuin activators (resveratrol, NAD^+^ precursors); Senolytic agents (emerging); Autophagy inducers (AMPK activators, metformin)	★
Endocrine Cross-Talk	IS (↓ Klotho); Gut-derived (TMAO, PAGln, indole metabolites)	VDR signaling disruption; CaSR desensitization; FGF23 resistance; Klotho deficiency; PTH excess; Gut dysbiosis → ↑ uremic toxin production; Microbiota–metabolite–vascular axis	25(OH)D < 20 ng/mL; 1,25(OH)_2_D ↓; Klotho < 400 pg/mL; TMAO ↑; Dysbiotic microbiome (↓ butyrate, ↑ proteolytic bacteria); Secondary hyperparathyroidism	CKD-MBD with hormonal imbalance; Gut dysbiosis; Inadequate vitamin D; Resistant sHPT; Multi-organ uremic syndrome	Selective VDRAs (paricalcitol); Calcimimetics; Klotho restoration strategies; Microbiota modulation (prebiotics, probiotics); Iron repletion (↓ FGF23)	★★
Integrated Toxic Continuum	Multi-toxin accumulation (all classes)	Self-reinforcing loops: Inflammation ↔ Pi retention ↔ oxidative stress ↔ endocrine dysfunction; Loss of vascular resilience; System-wide metabolic reprogramming; Organ becomes self-toxic	Multiple derangements: ↑ Pi, ↑ FGF23, ↑ PTH, ↑ IS/PCS, ↑ CRP, ↓ Klotho, ↓ Fetuin-A; Extensive CAC (Agatston > 400); Multi-organ uremic manifestations	Advanced/end-stage CKD; Multiple comorbidities; Severe CKD-MBD; Extensive vascular disease; Poor prognosis; “Point of no return” physiology	Multi-target combination therapy: Dialysis optimization + Pi control + antioxidants + VDRAs + anti-inflammatory agents; Future: Integrated precision protocols	★★

Notes: ★, ★★, ★★★ represent low, medium and high separately. ↓ indicates a decrease; ↑ indicates an increase.

**Table 2:** Biomarker Panel for Phenotypic Stratification and Therapeutic Monitoring in Uremic Toxin-driven Vascular Calcification.

Biomarker Category	Specific Biomarker	Measurement Method	Reference Range/Clinical Cut-off	Associated Phenotype(s)	Clinical Utility	Current Availability
**UREMIC TOXINS**						
Protein-bound solutes	Indoxyl sulfate (IS) ^‡^	HPLC, LC-MS/MS	Healthy: <5 μM; CKD 3–4: 20–80 μM; Dialysis: 50–200 μM; **High risk: >100 μM**	1 (Inflammatory-Oxidative), 3 (Epigenetic-Senescent)	Identifies oxidative/inflammatory phenotype; Marker of inadequate dialysis clearance; Correlates with CV events and mortality	Research labs; Specialized centers; Not routine clinical
Protein-bound solutes	p-Cresyl sulfate (PCS) ^‡^	HPLC, LC-MS/MS	Healthy: <10 μM; CKD 3–4: 30–100 μM; Dialysis: 80–300 μM; **High risk: >150 μM**	1 (Inflammatory-Oxidative), 5 (Integrated)	Synergistic with IS for vascular toxicity; Reflects gut dysbiosis; Predicts progression	Research labs; Limited clinical use
Gut-derived metabolites	TMAO (trimethylamine N-oxide) ^‡^	LC-MS/MS	Healthy: <5 μM; CKD: 10–50 μM; **High CV risk: >10 μM**	4 (Endocrine Cross-Talk), 1 (Inflammatory)	Marker of gut dysbiosis and platelet activation; Independent CV risk predictor; Therapeutic target (microbiome modulation)	Research labs; Emerging clinical availability
**MINERAL METABOLISM**						
Phosphate homeostasis	Serum phosphate (Pi) ^†^	Automated analyzer (colorimetric)	Normal: 2.5–4.5 mg/dL; CKD target: 3.5–5.5 mg/dL; **High risk: >5.5 mg/dL**	2 (Mineral-Metabolic)	Direct driver of VC; First-line therapeutic target; Guides phosphate binder therapy	Routine clinical; Widely available
FGF23-Klotho axis	Intact FGF23 ^‡^	ELISA (two-site immunoassay)	Normal: 10–50 RU/mL; CKD 3–4: 50–500 RU/mL; Dialysis: 500–5000+ RU/mL; **High risk: >200 RU/mL**	2 (Mineral-Metabolic), 4 (Endocrine)	Early marker of CKD-MBD; Predicts LVH, mortality; Reflects iron deficiency; Target for iron/HIF-PHDi therapy	Specialized labs; Increasing availability
FGF23-Klotho axis	Soluble Klotho ^§^	ELISA	Normal: 400–1000 pg/mL; CKD: 200–600 pg/mL; **Deficiency: <400 pg/mL**	2 (Mineral-Metabolic), 4 (Endocrine)	Anti-calcific defense marker; Inversely correlates with FGF23; Therapeutic restoration target	Research labs; Limited clinical
Parathyroid function	PTH (parathyroid hormone) ^†^	Automated immunoassay	Normal: 15–65 pg/mL; CKD 3–5 target: 2–9× ULN (KDIGO); **High risk: >600 pg/mL**	2 (Mineral-Metabolic), 4 (Endocrine)	Guides calcimimetic/VDRA therapy; Marker of bone turnover; Excess drives vascular calcification	Routine clinical; Widely available
**CALCIFICATION**						
MGP activation status	dp-ucMGP (dephosphorylated-uncarboxylated MGP) ^‡^	ELISA	Normal: <300 pmol/L; CKD: 500–2000 pmol/L; Dialysis: 1000–4000+ pmol/L; **High risk: >1500 pmol/L**	2 (Mineral-Metabolic), 3 (Epigenetic)	Marker of vitamin K deficiency and vascular calcification risk; Therapeutic target for vitamin K_2_; Predicts CV mortality	Specialized labs; Research use; Emerging clinical
Systemic inhibitor	Fetuin-A (α2-HS glycoprotein) ^§^	ELISA, nephelometry	Normal: 0.4–1.0 g/L; CKD/Dialysis: 0.2–0.6 g/L; **Deficiency: <0.3 g/L**	2 (Mineral-Metabolic), 5 (Integrated)	Low levels correlate with VC progression and mortality; Reflects inflammation and malnutrition (inverse acute-phase reactant)	Specialized labs; Research use
**INFLAMMATION & OXIDATIVE STRESS**						
Systemic inflammation	hs-CRP (high-sensitivity C-reactive protein) ^†^	Immunoturbidimetry	Low CV risk: < 1 mg/L; Intermediate: 1–3 mg/L; High: 3–10 mg/L; **Very high: >10 mg/L**	1 (Inflammatory-Oxidative), 5 (Integrated)	Marker of systemic inflammation; Predicts CV events; Reflects oxidative/inflammatory phenotype dominance	Routine clinical; Widely available
Pro-inflammatory cytokines	IL-6 (interleukin-6) ^§^	ELISA, multiplex assay	Normal: < 5 pg/mL; Elevated in CKD: 5–20 pg/mL; **High inflammatory state: >10 pg/mL**	1 (Inflammatory-Oxidative), 5 (Integrated)	Reflects NLRP3 activation and uremic inflammation; Correlates with mortality; Target for anti-inflammatory therapy	Specialized labs; Research use
**VASCULAR STRUCTURE & FUNCTION**						
Vascular calcification burden	CAC score (coronary artery calcium) ^†^	Non-contrast cardiac CT (Agatston score)	None: 0; Mild: 1–100; Moderate: 101–400; Severe: >400; **Very high risk: >1000**	All phenotypes (final common pathway)	Gold standard for VC quantification; Strong predictor of CV events and mortality; Guides therapeutic intensity	Widely available (CT required); Standard clinical imaging
Arterial stiffness	PWV (pulse wave velocity) ^‡^	Applanation tonometry, oscillometric devices	Normal: <7 m/s; Intermediate: 7–10 m/s; **High risk: >10 m/s**; Dialysis typical: 10–15 m/s	2 (Mineral-Metabolic), 5 (Integrated)	Functional marker of vascular stiffness; Predicts CV outcomes; Reflects cumulative vascular injury	Specialized centers; Research use; Emerging clinical availability
Endothelial function	FMD (flow-mediated dilation) ^§^	High-resolution ultrasound	Normal: >7%; Impaired: 3–7%; **Severe dysfunction: <3%**	1 (Inflammatory-Oxidative), 4 (Endocrine)	Marker of NO bioavailability and endothelial health; Reflects early vascular injury; Therapeutic monitoring for antioxidants	Specialized centers; Research use; Operator-dependent

Biomarker availability: routinely available in clinical laboratories (†), specialized centers only (‡), research-use only (§). Clinical feasibility discussed in text.

**Table 3:** Therapeutic Strategies for Vascular Calcification in Chronic Kidney Disease: Mechanisms, Evidence, and Clinical Translation. The following tables ((**A**)–(**E**)) present therapeutic interventions organized by phenotypic cluster. Each table includes mechanism of action, evidence level (Clinical/Investigational/Experimental), clinical trial data, current regulatory status, and major limitations.

**(A). Therapeutic Interventions for Inflammatory-Oxidative Phenotype**						
**Therapeutic Class**	**Agent/Intervention**	**Mechanism of Action**	**Evidence Level**	**Clinical Evidence**	**Current Status**	**Major Limitations**
**Intensified dialysis**	Hemodiafiltration/Expanded HD	Enhanced middle-molecule clearance; ↓ IS, ↓ PCS (20–30%)	Clinical	CONVINCE [78] ↓ mortality; Post hoc: greatest benefit in high IS/PCS tertiles	Clinical use	Facility availability; cost; patient tolerance
**Oral sorbents**	AST-120	Binds indole precursors; ↓ IS generation	Investigational	EPPIC-1/2 [79]: negative in unselected CKD; positive in high-IS subgroups	Approved (Japan); Not approved (US/EU)	Heterogeneous trial results; phenotype-enriched trials needed
**Antioxidants**	N-acetylcysteine	ROS scavenging; ↑ glutathione; ↓ NF-κB, NLRP3	Investigational	SPACE trials [80]: ↓ oxidative markers; no large VC-focused RCT	Off-label use	Limited clinical VC trials; high doses needed
**Antioxidants**	Resveratrol	SIRT1 activation; ↑ autophagy; ↓ ROS, NF-κB	Investigational	Small trials: ↓ CRP, ↑ endothelial function	Nutraceutical	Low bioavailability; limited large RCTs
**NLRP3 inhibitors**	MCC950, others	Selective NLRP3 inhibition; ↓ IL-1β; ↓ sterile inflammation	Experimental	Phase 2 in other diseases; preclinical ↓ VC in CKD models	Phase 2 (non-VC)	No VC-specific trials; safety profile being established
**AhR antagonists**	CH-223191	Blocks AhR activation by IS/PCS; ↓ NF-κB; ↓ osteogenic signaling	Experimental	Preclinical: ↓ IS-induced VC and endothelial dysfunction	Preclinical only	No human data; pharmacokinetics unclear
**(B). Therapeutic Interventions for Mineral-Metabolic Phenotype**						
**Therapeutic Class**	**Agent/Intervention**	**Mechanism of Action**	**Evidence Level**	**Clinical Evidence**	**Current Status**	**Major Limitations**
**Non-Ca phosphate binders**	Sevelamer	Binds intestinal Pi; ↓ Ca-Pi product; anti-inflammatory effects	Clinical	CARE-2 trial [81]: ↓ CAC progression vs Ca-based; INDEPENDENT [82]: trend ↓ mortality	Approved	GI intolerance; pill burden; does not lower FGF23
**Non-Ca phosphate binders**	Lanthanum carbonate	Binds intestinal Pi; minimal systemic absorption	Clinical	LANDMARK [83]: ↓ VC progression; lower pill burden	Approved	Rare lanthanum deposition; long-term safety data limited
**Iron-based binders**	Ferric citrate	Binds Pi; improves iron status; ↓ FGF23	Clinical	PA21 Study Group [84]: non-inferior to sevelamer; ↓ FGF23	Approved	GI side effects; cost
**Calcimimetics**	Cinacalcet/Etelcalcetide	CaSR activation; ↓ PTH, ↓ Ca, ↓ Pi	Clinical	EVOLVE [85]: trend ↓ CV events; PARADIGM [86]: ↓ FGF23	Approved	GI intolerance; hypocalcemia; modest mortality benefit
**Vitamin D receptor activators**	Paricalcitol	Selective VDR activation; ↓ PTH; anti-inflammatory	Clinical	PRIMO [87]: ↓ LVH in CKD 3–4; lower hypercalcemia vs calcitriol	Approved	Risk of hypercalcemia/hyperphosphatemia
**Crystal growth inhibitors**	SNF472 [77]	Binds hydroxyapatite surface; prevents crystal growth	Clinical	CaLIPSO [25,75,76]: ↓ CAC and aortic valve calcification in HD	Phase 3 completed; regulatory review	IV administration 3x/week; cost; limited to dialysis
**Vitamin K_2_**	Menaquinone-7	γ-carboxylation of MGP → active form; ↓ dp-ucMGP	Investigational	Westenfeld [88]: ↓ dp-ucMGP; Valkyrie Study [89]: ↓ PWV trend	Multiple phase 2–3 trials	Heterogeneous results; optimal dose unclear; warfarin interaction
**Enzyme replacement**	ENPP1-Fc (INZ-701)	Restores PPi generation from ATP; inhibits hydroxyapatite	Experimental	Preclinical: ↓ VC in CKD models; Phase 2 in rare disease	Phase 2; CKD trials planned	IV administration; cost; limited human CKD data
**TNAP inhibitors**	SBI-425	Selective TNAP inhibition; ↑ PPi/Pi ratio	Experimental	Preclinical: ↓ atherosclerotic calcification; preserves bone	Preclinical; Phase 1 planned	Human trials pending; potential bone effects
**Magnesium**	Oral/dialysate Mg	Competes with Ca^2+^; stabilizes amorphous Ca-Pi; ↑ Klotho	Investigational	RCTs ongoing: mixed results on ↓ CAC progression	Phase 3 trials ongoing	Hypermagnesemia risk; optimal target unclear
**(C). Therapeutic Interventions for Epigenetic-Senescent Phenotype**						
**Therapeutic Class**	**Agent/Intervention**	**Mechanism of Action**	**Evidence Level**	**Clinical Evidence**	**Current Status**	**Major Limitations**
**Vitamin K_2_**	Menaquinone-7	γ-carboxylation of MGP; limited efficacy once epigenetic silencing established	Investigational	Long-term dialysis trials: ↓ dp-ucMGP but limited ↓ VC progression	Multiple trials	Functional vitamin K resistance in epigenetic-senescent phenotype
**Senolytic agents**	Dasatinib + Quercetin	Selective elimination of p16^INK4a^-high senescent cells	Experimental	Preclinical: ↓ vascular senescence 50–70%; early human aging trials: safety shown	Phase 1–2 (aging); preclinical for CKD-VC	Very early for VC; no CKD-VC specific trials
**Senolytic agents**	Fisetin	Senescent cell clearance; ↓ SASP	Experimental	Preclinical aging models; early human trials ongoing	Phase 1–2	Very early stage; intermittent dosing unclear
**Autophagy enhancers**	Spermidine	Induces autophagy/mitophagy; ↓ calcifying vesicles; activates sirtuins	Experimental	Human aging trials: ↓ c ellular dysfunction; no CKD-VC RCT	Nutraceutical; Phase 2 (aging)	No VC-specific data; optimal dose unknown
**Autophagy enhancers**	Urolithin A	Mitophagy activation; mitochondrial renewal	Experimental	Preclinical: ↓ senescence markers; early human trials	Phase 1–2	Very early for VC; CKD trials needed
**NAD^+^ precursors**	NMN, Nicotinamide riboside	↑ NAD^+^ → SIRT1/3 activation; ↑ mitochondrial function	Experimental	Preclinical: ↓ VC, ↓ senescence; human trials early phase	Phase 1–2; nutraceutical	Very early for VC; optimal dose unclear; cost
**AMPK activators**	Metformin	AMPK activation; ↑ autophagy; ↓ mTOR; anti-inflammatory	Investigational	Observational CKD: ↓ CV events; preclinical: ↓ VC	Approved (diabetes); off-label CKD	Lactic acidosis risk; contraindicated eGFR <30
**SGLT2 inhibitors**	Empagliflozin, others	AMPK activation; ↑ autophagy; cardioprotective	Investigational	CV trials: ↓ CV events; preclinical: ↓ VC via autophagy	Approved (diabetes/HF/CKD)	VC effects indirect; dedicated VC trials lacking
**(D). Therapeutic Interventions for Endocrine Cross-Talk Phenotype**						
**Therapeutic Class**	**Agent/Intervention**	**Mechanism of Action**	**Evidence Level**	**Clinical Evidence**	**Current Status**	**Major Limitations**
**Vitamin D repletion**	Cholecalciferol/Calcifediol	Restore 25(OH)D levels; substrate for 1α-hydroxylation	Clinical	Observational: low 25(OH)D associated with ↑ VC; RCTs: mixed results	Clinical use	Optimal target level unclear; direct VC benefit uncertain
**VDRA**	Paricalcitol	VDR activation; ↓ PTH; anti-inflammatory; ↓ RAAS	Clinical	PRIMO [87]: ↓ LVH; pleiotropic CV effects	Approved	Hypercalcemia risk; narrow therapeutic window
**Iron supplementation**	Oral/IV iron	↑ Iron stores; ↓ HIF-1α; ↓ FGF23 transcription	Clinical	Meta-analyses [36]: Iron ↓ FGF23 20–30%; improves anemia	Clinical use for anemia	FGF23 reduction modest; iron overload risk
**HIF-PHD inhibitors**	Roxadustat, Daprodustat	Stabilize HIF → ↑ EPO; ↓ FGF23 indirectly	Clinical	Preclinical: ↓ FGF23 while treating anemia	Approved (Asia, EU); FDA review (US)	FGF23 lowering is secondary; CV safety debated
**Prebiotics**	Resistant starch, Inulin	Shift microbiota to saccharolytic; ↓ IS generation	Investigational	Small CKD trials: resistant starch ↓ IS 30%; ↑ microbiome diversity	Clinical use; ongoing trials	Limited large RCTs; optimal formulation unclear
**Probiotics**	*Bifidobacterium*, *Lactobacillus*	Restore beneficial bacteria; ↓ proteolytic metabolism	Investigational	Randomized trial [90]: modest ↓ IS, ↓ PCS; heterogeneous results	Clinical use	Strain-specific effects; standardization needed
**TMA production inhibitors**	3,3-dimethyl-1-butanol	Inhibits microbial TMA production from choline/carnitine	Experimental	Preclinical: ↓ TMAO; no human CKD trials	Preclinical	No clinical data; CKD trials needed
**Fecal microbiota transplant**	Donor microbiome	Restore healthy gut microbiome composition	Experimental	Case reports in CKD; no controlled VC trials	Investigational	Very early stage; safety and efficacy unknown in CKD
**(E). Therapeutic Interventions for Integrated Toxic Continuum Phenotype**						
**Therapeutic Class**	**Agent/Intervention**	**Mechanism of Action**	**Evidence Level**	**Clinical Evidence**	**Current Status**	**Major Limitations**
**Multi-target strategies**	Combination therapy	Simultaneous targeting of multiple pathways: mineral, inflammatory, cellular	Clinical	Standard practice: phosphate binders + calcimimetics + VDRA; no dedicated RCTs	Clinical use	Optimal combination unclear; no phenotype-guided protocols
**Intensified dialysis**	Extended hours/Increased frequency	Enhanced toxin clearance across all domains	Clinical	FHN trials: ↓ LVH with frequent HD; observational: ↓ mortality	Clinical use (limited)	Patient burden; vascular access; facility resources
**Crystal growth inhibitor**	SNF472	Universal anti-calcific; blocks terminal crystallization	Clinical	CaLIPSO [25,75,76]: ↓ CAC progression in advanced VC	Phase 3 completed; regulatory review	IV 3x/week; cost; maximal benefit in active crystallization
**Senolytic + anti-inflammatory**	Dasatinib-Quercetin + NLRP3 inhibitor	Eliminate senescent cells + suppress inflammasome	Experimental	Preclinical rationale strong; no human combination trials	Preclinical	Very early; safety and efficacy unknown
**Recombinant Klotho**	Soluble Klotho protein	Restore Klotho-mediated signaling; anti-aging effects	Experimental	Preclinical: ↓ VC, ↓ FGF23 toxicity; no human trials	Preclinical	Delivery route unclear; production challenges
**Kidney transplantation**	Living or deceased donor	Restores renal function; normalizes uremic milieu; most definitive intervention	Clinical	Observational: halts VC progression; modest regression in select cases	Standard of care	Donor availability; immunosuppression; not all patients eligible

↓ indicates a decrease; ↑ indicates an increase.

**Table 4:** Landscape of Clinical Trials Targeting Vascular Calcification in Chronic Kidney Disease.

Trial Name (ClinicalTrials.gov ID)	Intervention	Target Mechanism	Study Design	N (Enrolled/Target)	Primary Endpoint	Status/Key Results	Target Phenotype Cluster(s)
**CRYSTAL GROWTH INHIBITION**							
CaLIPSO (NCT02966028)	SNF472 (myo-inositol hexaphosphate) IV 3×/week	Hydroxyapatite crystal growth inhibition	Phase 3, RCT, double-blind, placebo-controlled	274 HD patients	Change in CAC and aortic valve calcification volume (CT) at 52 weeks	**Completed 2020:** SNF472 significantly slowed CAC progression (−55% relative difference, *p* < 0.05) and aortic valve calcification vs. placebo. Well-tolerated.	C2 (Mineral-Metabolic), C5 (Integrated)
CALCIPHYX (NCT04195906)	SNF472 IV during HD sessions	Hydroxyapatite crystal inhibition	Phase 3, open-label, single-arm	40 calciphylaxis patients	Complete wound healing at 12 weeks	**Completed 2022:** 47.5% achieved complete healing vs 21% historical controls. Significant pain reduction. SNF472 shows promise for calciphylaxis treatment.	2 (Mineral-Metabolic), C5 (Integrated)
**PHYSIOLOGICAL INHIBITOR RESTORATION**							
VitaVasK (NCT01742273)	Vitamin K_2_ (menaquinone-7) 360 μg/day oral × 12 months	MGP γ-carboxylation; inhibition of BMP2 signaling	Phase 2, RCT, open-label	53 HD patients	Change in CAC score (Agatston) and dp-ucMGP	**Completed 2012:** No significant ↓ in CAC score; significant ↓ dp-ucMGP. Trend for slowed progression in subgroup with baseline CAC < 400. Safe.	C2 (Mineral-Metabolic), C3 (Epigenetic)
VitaVasK (NCT01742273)	Vitamin K_2_ (menaquinone-7) 360 μg/day oral × 12 months	MGP γ-carboxylation; inhibition of BMP2 signaling	Phase 2, RCT, open-label	53 HD patients	Change in CAC score (Agatston) and dp-ucMGP	**Completed 2012:** No significant ↓ in CAC score; significant ↓ dp-ucMGP. Trend for slowed progression in subgroup with baseline CAC < 400. Safe.	C2 (Mineral-Metabolic), C3 (Epigenetic)
iPACK-HD (NCT04539418)	Vitamin K_2_ (menaquinone-7) vs. placebo	MGP activation; ↓ vascular stiffness	Phase 3, RCT, double-blind, placebo-controlled	80 HD patients	Change in PWV at 12 months	**Ongoing:** Expected completion 2025. Primary focus on arterial stiffness rather than calcification volume.	C2 (Mineral-Metabolic)
RenaKvit (NCT03303079)	Vitamin K_2_ (menaquinone-7) 360 μg/day vs. placebo	MGP carboxylation; anti-calcification	Phase 3, RCT, double-blind	90 CKD 3b-5 (non-dialysis)	Change in CAC progression at 12 months	**Completed 2020:** No significant difference in CAC progression between groups. Post hoc: possible benefit in subgroup with adequate vitamin K levels at baseline.	C2 (Mineral-Metabolic)
Magnesium Supplementation (NCT02542319)	Oral magnesium vs. placebo	Mg competes with Ca; stabilizes amorphous Ca-Pi; ↓ Wnt signaling	Phase 3, RCT, double-blind	148 CKD 3–4 patients	Change in CAC score at 12 months	**Completed 2019:** Trend for slower CAC progression in Mg group (not statistically significant). ↑ Serum Mg associated with ↓ CAC in subanalysis. Safe.	C2 (Mineral-Metabolic), 1 (Inflammatory)
**MINERAL METABOLISM MODULATION**							
EVOLVE (NCT00345839)	Cinacalcet vs placebo	CaSR activation; ↓ PTH, Ca, Pi	Phase 3, RCT, double-blind, placebo-controlled	3883 HD patients	Composite: death, MI, hospitalization for unstable angina, HF, or peripheral vascular event	**Completed 2012:** No significant reduction in primary composite endpoint (HR 0.93, *p* = 0.11). Post hoc and time-to-event analyses suggest potential CV benefit. Reduced hypercalcemia.	C2 (Mineral-Metabolic), C4 (Endocrine)
ADVANCE (NCT00345878)	Cinacalcet + low-dose vitamin D vs vitamin D alone	CaSR activation; ↓ PTH; improved Ca-Pi control	Phase 3, RCT, open-label	360 HD patients	Achievement of K/DOQI targets for PTH, Ca, Pi	**Completed 2005:** Cinacalcet group achieved targets more frequently. ↓ Ca, ↓ Pi, ↓ PTH vs. vitamin D alone. ↓ Incidence of hypercalcemia.	C2 (Mineral-Metabolic), C4 (Endocrine)
PARADIGM (NCT00977080)	Paricalcitol vs cinacalcet	Selective VDR activation vs. CaSR activation	Phase 3, RCT, open-label	72 HD patients	Change in LVH (cardiac MRI) and FGF23 at 48 weeks	**Completed 2014:** Both ↓ PTH similarly. Cinacalcet superior for ↓ FGF23. No difference in LVH regression. Paricalcitol associated with ↑ Ca/Pi.	C2 (Mineral-Metabolic), C4 (Endocrine)
**IRON-FGF23 AXIS INTERVENTION**							
Ferric Citrate Studies (Multiple)	Ferric citrate vs other phosphate binders	Pi binding; ↑ iron stores; ↓ FGF23; direct anti-calcific effects	Phase 3, multiple RCTs	>500 HD and CKD patients (various studies)	Pi control, iron parameters, FGF23 levels	**Completed 2015–2019:** Ferric citrate non-inferior for Pi control. ↑ Ferritin, ↓ FGF23 vs. non-iron binders. Preclinical data shows direct ↓ VSMC calcification and ↑ autophagy.	C2 (Mineral-Metabolic), C4 (Endocrine)
**DIALYSATE MODIFICATION**							
Low-Calcium Dialysate Trial (NCT07163936)	Dialysate Ca 1.25 mmol/L vs. 1.5 mmol/L	↓ Calcium loading during dialysis; ↓ positive Ca balance	Phase 2, RCT, crossover	50 HD patients	Change in serum Ca, PTH, and vascular stiffness (PWV)	**Ongoing:** Expected completion 2026. Hypothesis: Lower dialysate Ca may reduce Ca overload and slow VC without worsening bone disease.	C2 (Mineral-Metabolic)
**EMERGING MECHANISMS**							
ENPP1 Replacement (INZ-701)	ENPP1-Fc enzyme replacement IV	Restores PPi generation; inhibits hydroxyapatite	Phase 2, open-label, dose-ranging	~30 ENPP1 deficiency patients; CKD cohort planned	Safety, pharmacokinetics; exploratory: change in ectopic calcification	**Ongoing (Phase 2):** Approved for rare ENPP1 deficiency (GACI/ARHR2). Preclinical CKD data strong. CKD-specific trials in planning stages. Expected CKD trial initiation 2026.	C2 (Mineral-Metabolic)
TNAP Inhibitor Studies	SBI-425 (selective TNAP inhibitor)	Selective TNAP inhibition; ↑ PPi/Pi ratio; ↓ calcification	Preclinical Phase 1 planned	Animal models; human trial pending	Preclinical: ↓ atherosclerotic calcification, preserved bone	**Preclinical 2023:** Promising results in ApoE-/- mice. ↓ CAC, ↓ plaque burden. Bone architecture maintained. Phase 1 trial anticipated 2026–2027.	C2 (Mineral-Metabolic)

↓ indicates a decrease; ↑ indicates an increase. C2: Cluster 2; C3: Cluster 3; C4: Cluster 4; C5: Cluster 5.

## Data Availability

No new data were created or analyzed in this study.

## Abbreviations

AGEs, Advanced glycation end-products; Akt, Protein kinase B; ALP, Alkaline phosphatase; AMPK, AMP-activated protein kinase; ANK, Progressive ankylosis protein; AST-120, Spherical carbon adsorbent; ATF4, Activating transcription factor 4; ATP, Adenosine triphosphate; AhR, Aryl hydrocarbon receptor; BMP, Bone morphogenetic protein; CAC, Coronary artery calcium; CaSR, Calcium-sensing receptor; CHOP, C/EBP homologous protein; CKD, Chronic kidney disease; CKD-MBD, Chronic kidney disease-mineral and bone disorder; CPP, Calciprotein particles; CRP, C-reactive protein; DNA, Deoxyribonucleic acid; eIF2α, Eukaryotic initiation factor 2 alpha; ENPP1, Ectonucleotide pyrophosphatase/phosphodiesterase 1; EPO, Erythropoietin; ER, Endoplasmic reticulum; ERK, Extracellular signal-regulated kinase; EV, Extracellular vesicles; EndMT, FGF23, Fibroblast growth factor 23; FGFR1, Fibroblast growth factor receptor 1; FMD, Flow-mediated dilation; GGCX, Gamma-glutamyl carboxylase; GRP, Gla-rich protein; HD, Hemodialysis; HDAC, Histone deacetylase; HDF, Hemodiafiltration; HDx, Expanded hemodialysis; HIF, Hypoxia-inducible factor; HIF-PHI, Hypoxia-inducible factor prolyl hydroxylase inhibitor; IL, Interleukin; IS, Indoxyl sulfate; JNK, c-Jun N-terminal kinase; MAPK, Mitogen-activated protein kinase; MGP, Matrix Gla protein; miRNA, MicroRNA; Msx2, Msh homeobox 2; mTORC1, Mammalian target of rapamycin complex 1; NAD^+^, Nicotinamide adenine dinucleotide; NADPH, Nicotinamide adenine dinucleotide phosphate; NF-κB, Nuclear factor kappa B; NLRP3, NOD-like receptor family pyrin domain containing 3; NO, Nitric oxide; Nrf2, Nuclear factor erythroid 2-related factor 2; OPG, Osteoprotegerin; OPN, Osteopontin; PAGln, Phenylacetylglutamine; PCS, p-Cresyl sulfate; PERK, Protein kinase RNA-like endoplasmic reticulum kinase; PI3K, Phosphoinositide 3-kinase; PiT, Sodium-dependent phosphate transporter; PPi, Inorganic pyrophosphate; PTH, Parathyroid hormone; PWV, Pulse wave velocity; RAAS, Renin–angiotensin–aldosterone system; RANKL, Receptor activator of nuclear factor kappa-B ligand; ROS, Reactive oxygen species; Runx2, Runt-related transcription factor 2; SASP, Senescence-associated secretory phenotype; SCFA, Short-chain fatty acids; SGLT2, Sodium-glucose cotransporter 2; SIRT, Sirtuin; SM22α, Smooth muscle protein 22-alpha; SM-MHC, Smooth muscle myosin heavy chain; SNF472, Myoinositol hexaphosphate; SOD, Superoxide dismutase; TGF-β, Transforming growth factor beta; TLR, Toll-like receptor; TMA, Trimethylamine; TMAO, Trimethylamine N-oxide; TNAP, Tissue-nonspecific alkaline phosphatase; TNF-α, Tumor necrosis factor alpha; UVB, Ultraviolet B radiation; VC, Vascular calcification; VDR, Vitamin D receptor; VDRA, Vitamin D receptor activator; Vit-D3, Vitamin D3; VSMC, Vascular smooth muscle cell; dp-ucMGP, Dephosphorylated-uncarboxylated matrix Gla protein; hs-CRP, High-sensitivity C-reactive protein; sHPT, Secondary hyperparathyroidism; α-SMA, Alpha-smooth muscle actin.
